# Rabphilin 3A retains NMDA receptors at synaptic sites through interaction with GluN2A/PSD-95 complex

**DOI:** 10.1038/ncomms10181

**Published:** 2015-12-18

**Authors:** Jennifer Stanic, Mario Carta, Ivano Eberini, Silvia Pelucchi, Elena Marcello, Armando A. Genazzani, Claudia Racca, Christophe Mulle, Monica Di Luca, Fabrizio Gardoni

**Affiliations:** 1 DiSFeB, Dipartimento di Scienze Farmacologiche e Biomolecolari, Università degli Studi di Milano, Via Balzaretti 9, Milano 20133, Italy; 2 Institut Interdisciplinaire de Neurosciences, University of Bordeaux, CNRS UMR 5297, Bordeaux 33000, France; 3 Dipartimento di Scienze del Farmaco, Università degli Studi del Piemonte Orientale ‘Amedeo Avogadro', Novara 28100, Italy; 4 Institute of Neuroscience, Newcastle University, Newcastle upon Tyne NE2 4HH, UK

## Abstract

NMDA receptor (NMDAR) composition and synaptic retention represent pivotal features in the physiology and pathology of excitatory synapses. Here, we identify Rabphilin 3A (Rph3A) as a new GluN2A subunit-binding partner. Rph3A is known as a synaptic vesicle-associated protein involved in the regulation of exo- and endocytosis processes at presynaptic sites. We find that Rph3A is enriched at dendritic spines. Protein–protein interaction assays reveals that Rph3A N-terminal domain interacts with GluN2A(1349–1389) as well as with PSD-95(PDZ3) domains, creating a ternary complex. Rph3A silencing in neurons reduces the surface localization of synaptic GluN2A and NMDAR currents. Moreover, perturbing GluN2A/Rph3A interaction with interfering peptides in organotypic slices or *in vivo* induces a decrease of the amplitude of NMDAR-mediated currents and GluN2A density at dendritic spines. In conclusion, Rph3A interacts with GluN2A and PSD-95 forming a complex that regulates NMDARs stabilization at postsynaptic membranes.

## Abstract (web summary)

Rabphilin 3A is a synaptic vesicle-associated protein involved in membrane trafficking at presynaptic sites. Here Stanic *et al.* show that Rabphilin 3A binds to the NMDA receptor GluN2A and stabilises its surface localisation at postsynaptic sites in dendritic spines.

## Graphical abstract

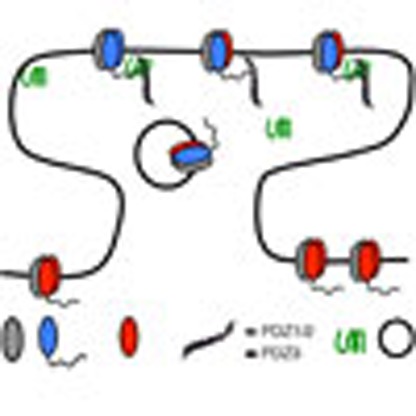

The functional properties of NMDARs (*N*-methyl-D-aspartate receptors) depend on their subunit composition. The identity of NMDAR GluN2-type subunits regulates biophysical and pharmacological properties of the receptor and influences receptor assembly, signalling and localization^1^. GluN2A-containing NMDARs are rather stable at synapses^2^,^3^. Conversely, GluN2B-containing NMDARs compose a more mobile pool of NMDARs present at both synaptic and extrasynaptic sites^4^,^5^. In addition, the subunit composition of synaptic NMDARs changes during development and after induction of activity-dependent synaptic plasticity^6^,^7^. During development, NMDARs switch from di-heteromes containing primarily GluN1-GluN2B to NMDARs enriched in GluN2A and composed of GluN1-GluN2A-GluN2B subunit^6^,^8^, this switch being under the control of epigenetic processes^9^.

It is believed that protein–protein interactions determine the specific synaptic retention of NMDARs with different GluN2-type subunits. In the last decade, PSD-95, Discs Large and Zona Occludens 1 (PDZ)-mediated interactions with PSD-MAGUK members, for example, PSD-95, have been clearly demonstrated to favour surface expression and stabilization of both GluN2A and GluN2B within the postsynaptic membrane^10^,^11^. A working model proposes that binding of GluN2 subunits to distinct PSD-MAGUKs plays a key role in NMDAR localization to either synaptic or extrasynaptic sites^1^,^7^. Current data support this scenario for GluN2B as the disruption of the GluN2B PDZ-binding domain results in a loss of synaptic GluN2B^12^,^13^. The mechanisms responsible for synaptic retention of NMDARs containing the GluN2A subunit are much less clear. Mice lacking the C-terminal domain of GluN2A show a reduced synaptic GluN2A expression^14^,^15^ and peptides affecting GluN2A/PSD-MAGUKs interaction reduce synaptic retention of NMDARs^3^,^16^. Notably, additional domains in the GluN2A C-terminus acting to stabilize NMDARs at synaptic sites have been proposed^17^,^18^,^19^. Based on these considerations, we performed a yeast two-hybrid (Y2H) screening using the intracellular C-terminal sequence of GluN2A and we identified Rabphilin 3A (Rph3A) as a potential partner.

Rph3A is known as a vesicle-associated presynaptic protein, first identified as a binding partner of Rab3A^20^, involved in the regulation of synaptic vesicle traffic^21^. Rph3A directly binds to other proteins including the MAGUK protein CASK^22^, SNAP-25 (ref. 23) and MyoVa^24^, localized in the pre- or post-synaptic compartment. Mice that lack Rph3A are viable and fertile without obvious physiological impairments^25^. Notably, synaptic properties impaired in Rab3A knock-out mice were found unaffected in Rph3A knock-out mice^25^. Thus, even if a large amount of information on the presynaptic pool of Rph3A is available, there is no consensus on the precise role of Rph3A in presynaptic terminals. And, no information is available on a possible postsynaptic localization and function of Rph3A.

Here we demonstrate the presence of Rph3A in the postsynaptic density (PSD) at excitatory synapses and indicate a novel function for Rph3A in the stabilization of GluN2A-containing NMDARs at synapses through the formation of a ternary complex with GluN2A and PSD-95.

## Results

### Rph3A is present at dendritic spines of excitatory synapses

**Figure 1:**
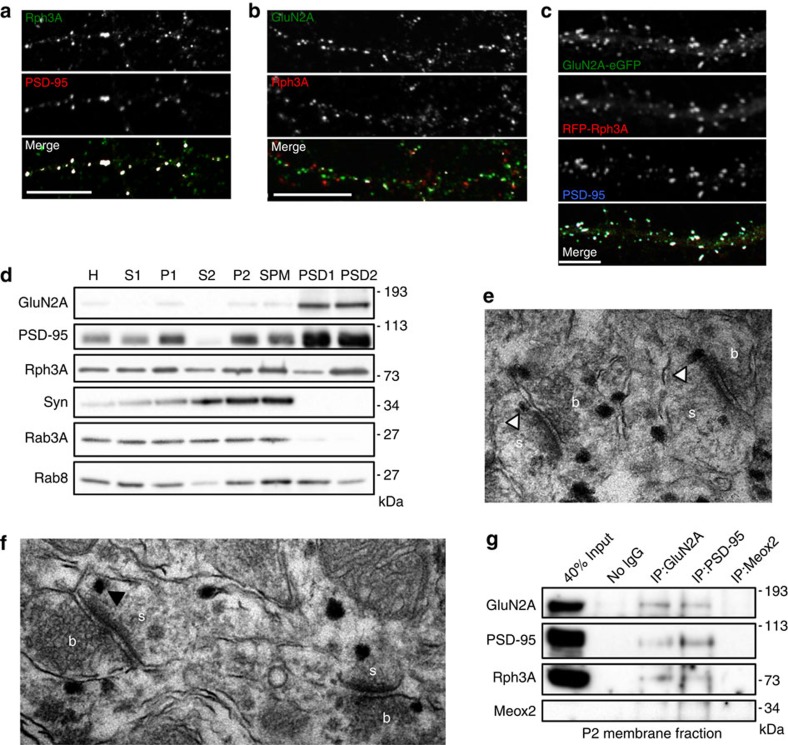
Subcellular distribution of Rph3A and interaction with GluN2A and PSD-95. (**a**) Fluorescence immunocytochemistry of Rph3A (green) and PSD-95 (red) in *DIV15* primary hippocampal neurons. On last panel (merge), co-localization points are shown in white. Scale bar, 10 μm. (**b**) Fluorescence immunocytochemistry of GluN2A (green) and Rph3A (red) in *DIV15* primary hippocampal neurons. On last panel (merge), co-localization points are shown in white. Scale bar, 10 μm. (**c**) Fluorescence immunocytochemistry of eGFP-GluN2A (green), RFP-Rph3A (red) and endogenous PSD-95 (blue) in *DIV15* primary hippocampal neurons transfected with eGFP-GluN2A and RFP-Rph3A (*DIV9*). Scale bar, 10 μm. (**d**) Subcellular expression of GluN2A, PSD-95, Synaptophysin (Syn), Rph3A, Rab3A and Rab8 in rat hippocampus. H, homogenate; S1/2, supernatant 1/2; P1/2, pellet 1/2; SPM, synaptosomal plasma membrane; PSD1/2, postsynaptic density fraction 1/2. (**e**,**f**) Immunolabelling of Rph3A in dendritic spines of pyramidal cells in the CA1 stratum radiatum of the hippocampus. Electron microscopy images show that Rph3A is found lateral to the PSD (white arrowheads) and at different positions in dendritic spines (white arrowheads). b, bouton, s, spine. (**g**) Co-immunoprecipitation experiments on rat hippocampal P2 fractions using polyclonal GluN2A, monoclonal PSD-95 and irrelevant monoclonal Meox2 antibodies show that Rph3A is associated with both GluN2A and PSD-95.

A Y2H screening, performed using the GluN2A C-terminal domain (amino acids (aa) 839–1,461, thus lacking the PDZ-binding sequence) as bait, identified Rph3A as a novel GluN2A-binding protein (see Methods for details). Because Rph3A has been extensively described as a presynaptic protein^21^, we first verified whether Rph3A was also present in the postsynaptic compartment where GluN2A resides. Fluorescent immunocytochemistry of primary hippocampal neurons showed that Rph3A co-localizes with PSD-95 and GluN2A along dendrites as shown in double (Fig. 1a,b) and triple immunostaining experiments (Supplementary Fig. 1a). Interestingly, Rph3A co-localization with GluN2A was detected only in PSD-95-positive postsynaptic clusters. In addition, Rph3A/PSD-95 co-localization was observed along MAP2-positive dendrites (Supplementary Fig. 1b). Similarly, RFP-Rph3A and eGFP-GluN2A expressed in hippocampal neurons showed a high degree of co-localization at PSD-95-positive clusters (Fig. 1c). As expected, Rph3A was also found to co-localize with the presynaptic interacting protein Rab3A^20^ (Supplementary Fig. 1c) as well as with the pre-synaptic marker Bassoon (Supplementary Fig. 1d). Using a biochemical fractionation approach to isolate purified excitatory PSDs from rat hippocampus, we found the presence of Rph3A in all subcellular fractions. Rph3A was enriched in synaptosomal plasma membranes and PSD2 fractions (Fig. 1d) similar to GluN2A and PSD-95, which are known as main PSD proteins. As internal controls, synaptophysin and Rab3A, two strictly presynaptic proteins, were present in several subcellular compartments analysed but not in PSD1 and PSD2 purified fractions (Fig. 1d). Rab8, a small GTPase that is specifically involved in the regulation of secretory/recycling vesicles, shows a widespread distribution in all synaptic compartments (Fig. 1d). In agreement with biochemical data, electron microscopic analysis showed the presence of Rph3A not only in the presynaptic bouton but also at the lateral domain of the PSD (Fig. 1e) as well as at multiple locations within dendritic spines and along dendritic shafts (Fig. 1f). Overall, these results indicate that Rph3A is present both at presynaptic and postsynaptic sites.

### Rph3A interacts with GluN2A and with PSD-95

Several experimental approaches were used to substantiate the Y2H data and to confirm the interaction between Rph3A and GluN2A. Rph3A/GluN2A interaction was detected in co-immunoprecipitation (co-IP) experiments from rat hippocampal P2 crude membrane fractions (Fig. 1g). Anti-GluN2A immunoprecipitated samples from P2 fractions were analysed in western blotting (WB) with Rph3A, GluN2A, PSD-95 and with the nuclear protein Meox2 as a control. We found a specific interaction of GluN2A with PSD-95 and Rph3A (Fig. 1g). Interestingly, both GluN2A and Rph3A also interacted with PSD-95 (Fig. 1g). These interactions are specific, as no signal for GluN2A, PSD-95 or Rph3A was obtained by using the irrelevant anti-Meox2 antibody or in the absence of the antibody in the co-IP assay (Fig. 1g).

**Figure 2:**
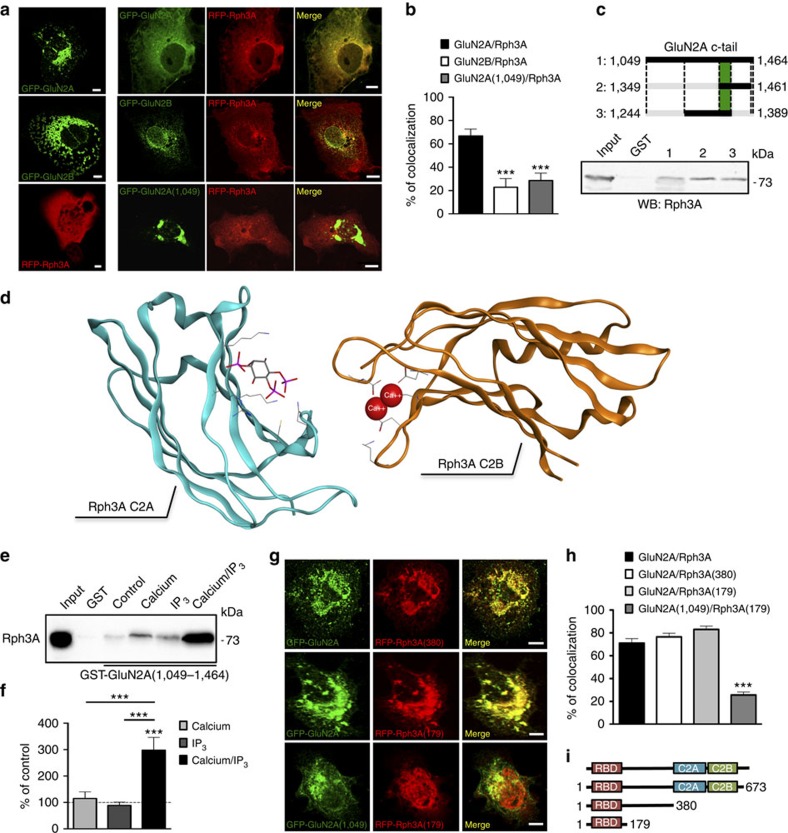
Rph3A interacts with GluN2A. (**a**) Confocal imaging of COS7 cells transfected with eGFP-GluN2A (green), eGFP-GluN2B (green) or RFP-Rph3A (red) and cells co-transfected with either eGFP-GluN2A, eGFP-GluN2B and RFP-Rph3A or eGFP-GluN2A(1,049) (green) and RFP-Rph3A. Scale bars, 10 μm. (**b**) Bar graph representing the percentage of co-localization between eGFP-GluN2A and RFP-Rph3A, eGFP-GluN2B and RFP-Rph3A, eGFP-GluN2A(1,049) and RFP-Rph3A. Data are presented as mean±s.e.m., *n*=5–9, ***P<0.001, unpaired Student's *t*-test. (**c**) GST pull-down assay of Rph3A using C-terminal GluN2A GST fusion protein with different sizes on rat brain extracts. 1, GluN2A (1,049–1,464); 2, GluN2A (1,349–1,461); 3, GluN2A (1,244–1,389). (**d**) Tridimensional model for the topological arrangement of the C2 domains of Rph3A (C2A in cyan and C2B in orange), modelled on the C2 domains of rat synaptotagmin-3. Each domain can bind both IP_3_ and 2 calcium ions; however, in the selected crystallographic structures, IP_3_ (stick rendering and CPK colouring) was solved only with the C2A and calcium (red spheres) with the C2B domain. The topological arrangement of the Rab-binding domain with respect to the C2 domains cannot be accurately modelled. (**e**,**f**) GST pull-down assay of Rph3A using C-terminal GluN2A(1,049–1,464) GST fusion protein in the presence or absence of calcium (20 mM) and IP_3_ (1 mM). The bar graph represents the binding of Rph3A to the fusion protein expressed as percentage of control (in absence of both calcium and IP_3_). Data are presented as mean±s.e.m., *n*=6, ***P<0.001; one-way ANOVA followed by Tukey *post-hoc* test. (**g**) Confocal imaging of COS7 cells co-transfected with eGFP-GluN2A and RFP-Rph3A(380) (upper panels), eGFP-GluN2A and RFP-Rph3A(179) (middle panels) or eGFP-GluN2A(1,049) and RFP-Rph3A(179) (lower panels). Scale bars, 10 μm. (**h**) Bar graph representing the percentage of co-localization between GluN2A and RFP-Rph3A constructs. Data are presented as mean±s.e.m., *n*=10, ***P<0.001; one-way ANOVA followed by Tukey *post-hoc* test, GluN2A(1,049)/Rph3A(179) versus GluN2A/Rph3A, GluN2A(1,049)/Rph3A(179) versus GluN2A/Rph3A(380), GluN2A(1,049)/Rph3A(179) versus GluN2A/Rph3A(179). (**i**) Representative schematic of the different mouse Rph3A mutant constructs used.

Co-clustering assay in heterologous COS7 cells and pull-down experiments were used to identify GluN2A, Rph3A and PSD-95 domains involved in the binding. As previously described^26^, when transfected in the absence of GluN1 subunit, eGFP-GluN2A or eGFP-GluN2B subunits displayed a clustered perinuclear distribution (Fig. 2a, left panels). Co-transfection of RFP-Rph3A with eGFP-GluN2A caused a redistribution of eGFP-GluN2A throughout the cell (Fig. 2a, right panels). Conversely, no effect on eGFP-GluN2B clustered distribution was observed when co-transfected with RFP-Rph3A (Fig. 2a, right panels). In addition, quantification of eGFP-GluN2A co-clustering with RFP-Rph3A showed a high co-localization value when compared with eGFP-GluN2B/RFP-Rph3A-transfected cells (Fig. 2b, ***P=0.0003, unpaired Student's *t*-test). Co-transfection of RFP-Rph3A with a mutant plasmid of GluN2A with a stop codon inserted at the aa 1,049 position fused with enhanced Green Fluorescent Protein (eGFP) (eGFP-GluN2A(1,049); Fig. 2a) revealed a significant decrease in the co-localization of GluN2A and Rph3A (Fig. 2b, ***P=0.0006, unpaired Student's *t*-test), thus indicating that the distal GluN2A(1,049–1,464) C-terminal domain is needed for the interaction with Rph3A. Glutathione *S*-transferase (GST) pull-down assay of Rph3A with fusion proteins of GluN2A C-tails of various lengths identified the region comprised between aa 1,349 and aa 1,389 as responsible for the interaction with Rph3A (Fig. 2c). Notably, no sequence homology was found between the GluN2A(1,349–1,389) sequence and C-tail domains of all other NMDAR and α-amino-3-hydroxy-5-methyl-4-isoxazolepropionic acid receptor (AMPAR) subunits, further confirming the specificity of Rph3A/GluN2A interaction.

Rph3A is an extrinsic membrane protein^20^,^27^, able to bind, through both its C2 domains (C2A (384–488 aa) and C2B (542–645 aa)), IP_3_ in a Ca^2+^-dependent manner. The calcium-bound C2 domains mediate interactions with phospholipid bilayers. Notably, IP_3_ and Ca^2+^ binding to the C2A domain mutually enhance each other. In particular, it has already been described, through both computational and experimental data, that the presence of Ca^2+^ ions induces a conformational rearrangement of a specific Rph3A loop (namely, CBL3), which is involved in IP_3_ binding^28^,^29^. Even if no crystallographic structure is available for rat Rph3A as a whole, its above-described domains have been separately solved. For this reason, we propose (see Fig. 2d for details) a relative arrangement of the two crystallized Rph3A C2 domains (PDB ID: 4NP9 for C2A and 2CM5 for C2B)^29^ based on the crystallographic structure of the C2 domains (A and B) of rat synaptotagmin-3 (PDB ID: 1DQV)^30^. Each Rph3A C2 domain binds two Ca^2+^ ions and one IP_3_ molecule. In agreement with these observations, Ca^2+^ or IP_3_ alone did not modify the affinity of Rph3A for GluN2A C-tail in a pull-down assay (Fig. 2e,f and Supplementary Fig. 2a; ***P<0.001, one-way analysis of variance (ANOVA) followed by Tukey *post-hoc* test). Conversely, co-incubation of samples with Ca^2+^ and IP_3_ induce a dramatic increase of Rph3A binding to GluN2A (Fig. 2e,f), confirming that both Ca^2+^ and IP_3_ are necessary for an efficient molecular recognition mechanism.

Rat Rph3A contains three main domains (see Fig. 2i for details): the N-terminal Rab-binding domain (residues 40–157)^31^,^32^,^33^ and two C-terminal C2-like domains (see above)^27^,^31^,^33^. It also contains a FYVE-type zinc finger (residues 88–145), which targets Rph3A to cell membranes through the highly specific interaction with the membrane lipid phosphatidylinositol-3-phosphate. RFP-Rph3A-truncated mutants were prepared and co-transfected with eGFP-GluN2A in COS-7 cells in order to identify the Rph3A domain responsible for the interaction with GluN2A. Deletion of C2A and C2B domain of Rph3A as in Rph3A(380) construct did not interfere with GluN2A/Rph3A clustering (Fig. 2g–i). Similarly, Rph3A(179) construct bearing a stop codon at aa 179 still interacted with GluN2A as demonstrated by co-localization assay thus indicating that the N-terminal Rab-binding domain is responsible for the binding with GluN2A (Fig. 2g,h). As expected, GFP-GluN2A(1,049) failed to interact with RFP-Rph3A(179) (Fig. 2g,h, ***P<0.001; one-way ANOVA followed by Tukey *post-hoc* test).

**Figure 3:**
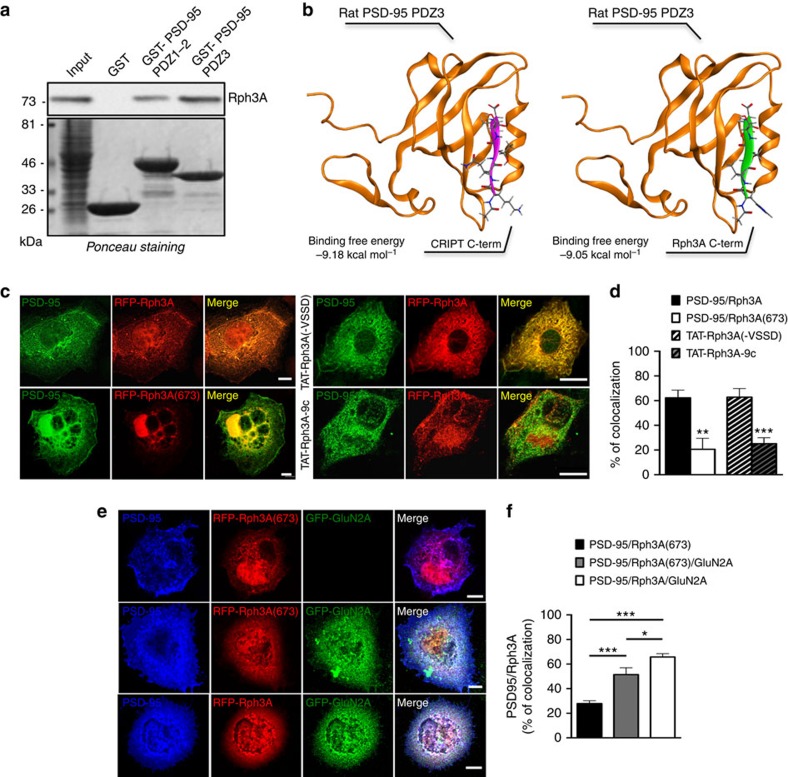
Rph3A interacts with PSD-95. (**a**) GST pull-down assay of Rph3A using PSD-95 PDZ1–2 or PSD-95 PDZ3 GST fusion proteins on rat brain extracts. Upper panel: WB analysis performed by using Rph3A antibody. Lower panel: Ponceau staining showing the amount of proteins loaded in each lane. (**b**) Left panel: complex between the PDZ3 domain of the rat PSD-95 (in orange) and the CRIPT protein C-terminus (in purple); right panel: complex between the PDZ 3 domain of the rat PSD-95 (in orange) and the Rph3A C-terminus (in green). Proteins are rendered as ribbons, peptides both as ribbons and sticks (with CPK colouring). The beta-sheet augmentation, typical of the PDZ domain molecular recognition mechanism, can be appreciated in both complexes. Binding free energies are reported for each complex. (**c**) Confocal imaging of COS7 cells co-transfected with PSD-95 (green) and RFP-Rph3A (red) in the presence or absence of TAT-Rph3A-9c or TAT-Rph3A(−VSSD), or PSD-95 and RFP-Rph3A(673) (red). Scale bars, 10 μm. (**d**) Bar graph representing the percentage of co-localization between PSD-95 and RFP-Rph3A in the presence or absence of TAT-Rph3A-9c or TAT-Rph3A(−VSSD), and PSD-95 and RFP-Rph3A(673) (*n*=5–10; unpaired Student's *t* test). (**e**) Confocal imaging of COS7 cells co-transfected with PSD-95 (blue), RFP-Rph3A or RFP-Rph3A(673) (red) in the presence or absence of GFP-GluN2A (green). Scale bar, 10 μm. (**f**) The bar graph represents the percentage of co-localization between PSD-95 and RFP-Rph3A constructs (*n*=8; one-way ANOVA followed by Tukey *post-hoc* test). All data are presented as mean±s.e.m., *P<0.05, **P<0.01, ***P<0.001.

Rph3A contains at its C-terminus a sequence (HVSSD) that resembles a putative PDZ-binding motif and previous studies indicated that Rph3A interacts with the presynaptic MAGUK protein CASK^22^. GST pull-down assays using GST fusion proteins of the PDZ1–2 and PDZ3 domains of PSD-95 revealed that Rph3A can bind PDZ1–2 and PDZ3 domains of PSD-95, with a higher affinity for PDZ3 (Fig. 3a). To clarify the interaction of the C-terminus of rat Rph3A with the PDZ3 domain of rat PSD-95, molecular modelling tasks were carried out. A crystallographic structure of PSD-95 complexed with a peptide containing a PDZ-binding sequence was available^34^. PDZ-binding sequences usually consist of a signature motif ([FYST]-X-[FVA]), occurring at the very C-terminus of the target proteins. As the C-terminus of Rph3A is characterized by the HVSSD sequence, and does not exactly match the PDZ-binding signature, we computed the binding free energies of the Rph3A::PSD-95 PDZ3 complex and compared its value with that from PSD-95 PDZ3 complexed with its cognate ligands, that is, the C-terminus of the CRIPT protein. The primary structure of the CRIPT peptide, co-crystallized with PSD-95, was mutated *in silico* into HVSSD. The structures of the two complexes, one between PSD-95 PDZ3 and the peptide from CRIPT, and the second one between PSD-95 PDZ3 and the Rph3A C-terminus were then optimized and the binding free energies were measured. As shown in Fig. 3b, despite its incomplete compliance with the signature motif of the PDZ-binding sequence, the affinity of rat Rph3A C-terminus for PSD-95 PDZ3 (−9.05 kcal mol^−1^) is highly comparable with the affinity of a conventional PDZ-binding peptide (from CRIPT) for the same PSD-95 PDZ 3 domain (−9.18 kcal mol^−1^).

In agreement with the co-precipitation (Fig. 1g), pull-down (Fig. 3a) and molecular modelling studies (Fig. 3b), RFP-Rph3A displayed a high degree of co-localization with PSD-95 in co-transfected COS7 cells (Fig. 3c,d). We designed a cell-permeable TAT peptide containing the last 9 aa of Rph3A C-tail (TAT-Rph3A-9c) to compete with Rph3A for the binding to PSD-95. The TAT-Rph3A(−VSSD) peptide, lacking the PDZ-interaction domain (VSSD), was used as a control (Fig. 3c,d). TAT-Rph3A-9c significantly reduced the degree of PSD-95/RFP-Rph3A co-localization when compared with cells treated with the control peptide (Fig. 3c,d; ***P=0.0006; unpaired Student's *t*-test). Further, this co-localization was markedly decreased when co-transfecting PSD-95 with RFP-Rph3A(673), a mutant lacking the last 9 aa of Rph3A C-tail containing the PDZ-binding motif (Fig. 3c,d; **P=0.0014; unpaired Student's *t*-test), confirming the importance of this domain for the binding to PSD-95.

Notably, co-transfection of GluN2A was sufficient to induce a significant increase in PSD-95 co-localization degree also with RFP-Rph3A(673) (Fig. 3e,f; ***P<0.001; one-way ANOVA followed by Tukey *post-hoc* test). Interestingly, in the presence of GluN2A, PSD-95/Rph3A co-localization was significantly higher compared with PSD-95/Rph3A(673) thus suggesting a more efficient formation of the GluN2A/PSD-95/Rph3A ternary complex (Fig. 3e,f; *P<0.05, ***P<0.001; one-way ANOVA followed by Tukey *post-hoc* test).

### Rph3A stabilizes synaptic GluN2A by blocking endocytosis

The results described above indicate the presence of a GluN2A/Rph3A/PSD-95 ternary complex in the postsynaptic compartment. To understand the role of Rph3A interaction with GluN2A and PSD-95, we first silenced Rph3A expression in hippocampal neurons by means of a specific short hairpin RNA (shRNA; tGFP-shRph3A). tGFP-shRph3A validation was performed in COS7 cells transfected with RFP-Rph3A (Supplementary Fig. 2b) as previously reported^35^.

**Figure 4:**
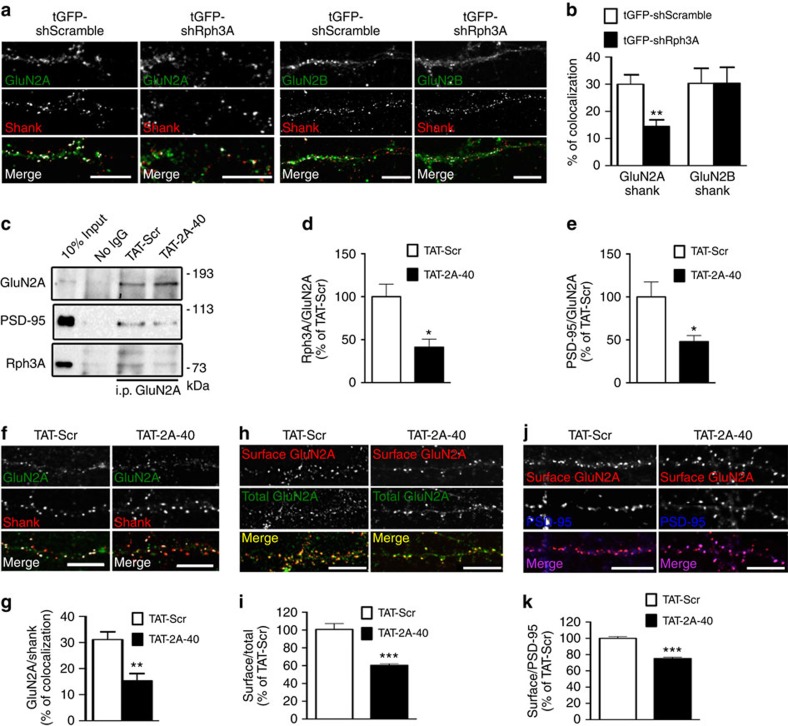
Effect of GluN2A/Rph3A complex on GluN2A synaptic availability in hippocampal neurons. (**a**) Fluorescence immunocytochemistry of GluN2A (green) and Shank (red) or GluN2B (green) and Shank in *DIV15* neurons transfected with tGFP-shScramble or tGFP-shRph3A (*DIV9*). (**b**) Bar graph representing the percentage of co-localization of GluN2A or GluN2B with Shank (*n*=10–19). (**c**–**e**) Co-immunoprecipitation of GluN2A with PSD-95 and Rph3A in P2 fractions from primary hippocampal neurons (*DIV15*) treated with TAT-2A-40 10 μM 30 min, showing a reduction of the interaction compared with animals treated with the control peptide TAT-Scr. The bar graphs show Rph3A/GluN2A (**d**) and PSD-95/GluN2A (**e**) co-immunoprecipitation expressed as % of TAT-Scr (*n*=4). (**f**) Fluorescence immunocytochemistry of GluN2A (green) and Shank (red) in *DIV15* neurons treated with TAT-Scr or 10 μM TAT-2A-40 for 30 min. (**g**) Bar graph representing the percentage of co-localization of GluN2A with Shank (*n*=7–14). (**h**) Fluorescence immunocytochemistry of surface GluN2A (red) and total GluN2A (green) in *DIV15* hippocampal neurons treated for 30 min with 10 μM TAT-Scr or TAT-2A-40. (**i**) Bar graph representation of the percentage of integrated density ratio GluN2A surface/total compared with the mean of TAT-Scr (*n*=119–144). (**j**) Fluorescence immunocytochemistry of surface GluN2A (red) and PSD-95 (blue) in *DIV15* hippocampal neurons treated for 30 min with TAT-Scr or TAT-2A-40 10 μM. (**k**) Bar graph representation of the percentage of integrated density ratio surface GluN2A/PSD-95 compared with the mean of TAT-Scr (*n*=119–150). All data are presented as mean±s.e.m.; *P<0.05, **P<0.01, ***P<0.001; unpaired Student's *t* tests. All scale bars represent 10 μm.

To isolate the postsynaptic effect of shRph3A, only transfected neurons very distant from one another, with no contact, were considered for quantitative analysis. The synaptic localization of GluN2A (*DIV15*; GluN2A/Shank co-localization) was decreased in primary hippocampal neurons transfected with tGFP-shRph3A (at *DIV9*) when compared with neurons transfected with tGFP-shScramble (Fig. 4a,b; **P=0.0011; unpaired Student's *t*-test). No difference was found in the synaptic localization of GluN2B (Fig. 4a,b; *P*=0.9951; unpaired Student's *t*-test).

To confirm these results and to investigate the role of the GluN2A/Rph3A/PSD-95 complex at the postsynaptic site, we used cell-permeable TAT peptides (CPPs) able to interfere with these protein–protein interactions in neurons. First, we characterized a TAT peptide containing the GluN2A(1,349–1,389) domain responsible for GluN2A interaction with Rph3A (see Fig. 2c for details; TAT-2A-40); a scramble sequence was used as control peptide (TAT-Scr). Administration of TAT-2A-40 (10 μM, 30 min) to DIV14 primary hippocampal neurons led to a significant decrease of GluN2A/Rph3A interaction compared with TAT-Scr peptide in co-IP assays (Fig. 4c,d, *P<0.05; unpaired Student's *t*-test). Notably, disruption of GluN2A/Rph3A complex by TAT-2A-40 peptide led also to a concomitant decrease of GluN2A/PSD-95 interaction (Fig. 4c,e, *P<0.05; unpaired Student's *t*-test), thus further indicating the presence of a GluN2A/PSD-95/Rph3A ternary complex. As a control, we verified that TAT-2A-40 did not induce any alteration of GluN2A, GluN2B, PSD-95 and Rph3A protein levels as evaluated by WB analysis in neuronal homogenates (Supplementary Fig. 3a,b).

We observed a significant reduction in the degree of co-localization between GluN2A and the post-synaptic marker Shank in neurons treated with TAT-2A-40 compared with those treated with TAT-Scr (Fig. 4f,g; **P=0.0012; unpaired Student's *t*-test), indicating a decreased enrichment of GluN2A at synapses. This effect was specific for GluN2A, as TAT-2A-40 had no effect on GluN2B/Shank co-localization (Supplementary Fig. 3c,d; *P*=0.6568; unpaired Student's *t*-test). Furthermore, the relative expression of GluN2A at the membrane surface was significantly reduced in *DIV15* primary hippocampal neurons exposed to TAT-2A-40 compared with TAT-Scr (Fig. 4h,i; ***P<0.0001; unpaired Student's *t*-test), whereas no effect was observed on the surface expression of GluN2B (Supplementary Fig. 3e,g; *P*=0.1683; unpaired Student's *t*-test) or of the GluA1 subunit of AMPARs (Supplementary Fig. 3f,h; *P*=0.4062; unpaired Student's *t*-test). Similar results were obtained by quantifying the GluN2A surface expression within PSD-95 postsynaptic clusters showing that this reduction is independent of any possible alteration of PSD-95-positive dendritic spines (Fig. 4j,k; ***P<0.0001; unpaired Student's *t*-test).

**Figure 5:**
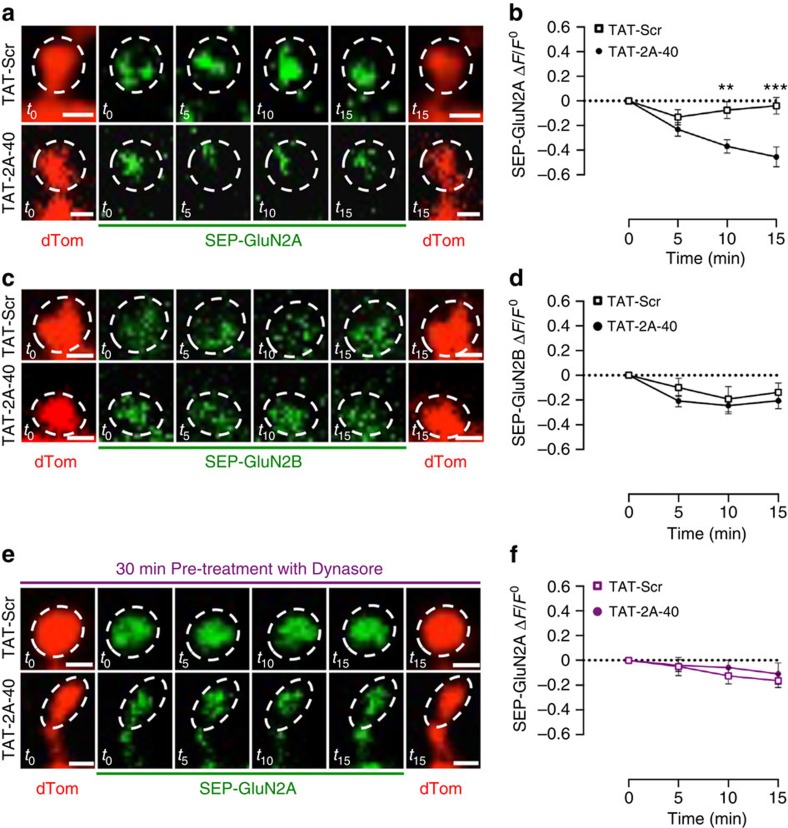
Effect of GluN2A/Rph3A complex on SEP-GluN2A membrane expression in spines of hippocampal neurons. (**a**) Live imaging of *DIV16* hippocampal neurons co-transfected with SEP-GluN2A (green) and dTom (red) after 0 min (*t*_0_), 5 min (*t*_5_), 10 min (*t*_10_) and 15 min (*t*_15_) of treatment with 10 μM TAT-Scr or TAT-2A-40. Scale bars, 1 μm. (**b**) *XY* graph representing the Δ*F*/*F*_0_ of SEP-GluN2A over time. Data are presented as mean±s.e.m., *n*=17–22, **P<0.01 and ***P<0.001; unpaired Student's *t*-test. (**c**) Live imaging of *DIV16* hippocampal neurons co-transfected with SEP-GluN2B (green) and dTom (red) after 0 min (*t*_0_), 5 min (*t*_5_), 10 min (*t*_10_) and 15 min (*t*_15_) of treatment with 10 μM TAT-Scr or TAT-2A-40. Scale bars, 1 μm. (**d**) *XY* graph representing the Δ*F*/*F*_0_ of SEP-GluN2B over time. Data are presented as mean±s.e.m., *n*=19. (**e**) Live imaging of pre-treated with Dynasore 80 μM for 30 min *DIV16* hippocampal neurons co-transfected with SEP-GluN2A (green) and dTom (red) after 0 min (*t*_0_), 5 min (*t*_5_), 10 min (*t*_10_) and 15 min (*t*_15_) of treatment with 10 μM TAT-Scr or TAT-2A-40. Scale bars, 1 μm. (**f**) *XY* graph representing the Δ*F*/*F*_0_ of SEP-GluN2A over time. Data are presented as mean±s.e.m., *n*=19–20.

We further investigated GluN2A surface levels over-time by performing time-lapse imaging of SEP-GluN2A and SEP-GluN2B constructs at glutamatergic synapses. A significant reduction in the signal for SEP-GluN2A, but not for SEP-GluN2B, was detected at synapses starting 10 min after TAT-2A-40 treatment (Fig. 5a,b, *t*_5_: *P*=0.2363; *t*_10_: **P=0.0023; *t*_15_: ***P=0.0003; unpaired Student's *t*-test). No difference in dendritic spine size between the two groups was observed within this time period (Fig. 5a). No effect of TAT-2A-40 was observed on SEP-GluN2B fluorescence (Fig. 5c,d). To evaluate whether these effects were correlated with an increase of GluN2A endocytosis, we repeated live-imaging experiments in the presence of the dynamin inhibitor dynasore^35^. Pre-treatment with dynasore (80 μM) completely abolished the observed reduction in SEP-GluN2A signal after TAT2A-40 treatment (Fig. 5e,f). These results highlight a selective role of Rph3A in the synaptic retention of GluN2A, and suggest that disruption of the GluN2A/Rph3A complex promotes GluN2A internalization.

### PSD-95/Rph3A interaction is required for GluN2A retention

**Figure 6:**
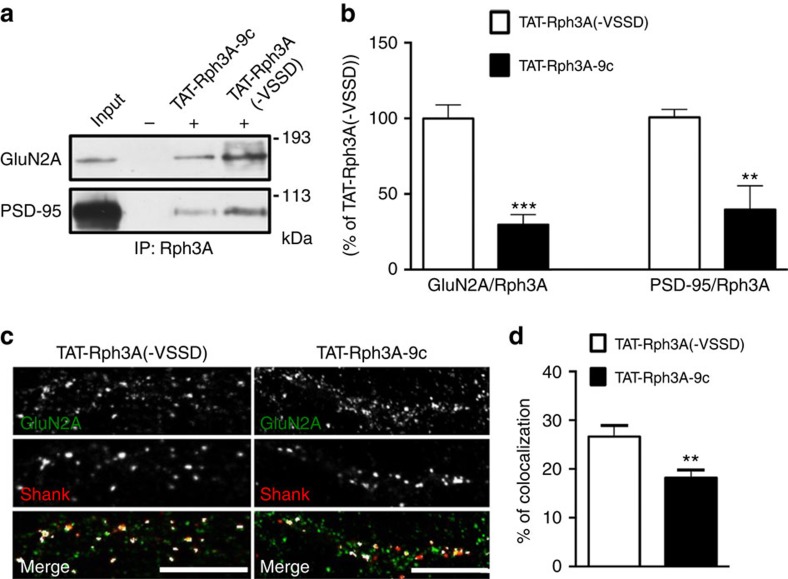
Effect of PSD-95/Rph3A complex on GluN2A synaptic availability in hippocampal neurons. (**a**–**c**) Co-immunoprecipitation of Rph3A with GluN2A and PSD-95 from P2 fraction from forebrain of mice 2 h after injection with TAT-Rph3A-9c (3 nmol g^−1^, i.p.) showing a reduction of both interactions compared with mice treated with the control peptide TAT-Rph3A(−VSSD). The bar graphs show GluN2A/Rph3A (left columns) and PSD-95/Rph3A (right columns) co-immunoprecipitation expressed as % of TAT-Rph3A(−VSSD); **P<0.01, *n*=3; ***P<0.001, *n*=5; unpaired Student's *t*-test. (**c**) Fluorescence immunocytochemistry of GluN2A (green) and Shank (red) in DIV15 neurons treated with 10 μM TAT-Rph3A(−VSSD) or TAT-Rph3A-9c for 30 min. Scale bars, 10 μm. (**d**) Bar graph representing the percentage of co-localization of GluN2A with Shank. Data are presented as mean±s.e.m., *n*=10, **P<0.01; unpaired Student's *t*-test.

We used TAT-Rph3A-9c peptide (see Fig. 3c,d for details) to understand the role of Rph3A/PSD-95 interaction in the formation of GluN2A/Rph3A/PSD-95 ternary complex and, consequently, in the regulation of GluN2A surface level at synapses. Rph3A co-IP with both GluN2A and PSD-95 in forebrain membrane fractions was reduced in mice treated with the TAT-Rph3A-9c peptide 1 h before killing (3 mol g^−1^ intraperitoneal (i.p.); Fig. 6a,b; ***P<0.001 GluN2A/Rph3A, **P<0.01 PSD-95/Rph3A; unpaired Student's *t*-test), thus suggesting that Rph3A/PSD-95 binding is needed for the stabilization of the GluN2A/PSD-95/Rph3A ternary complex. Accordingly, similar to what was described above for TAT-2A-40, treatment with TAT-Rph3A-9c led to a significant decrease of GluN2A levels at synaptic sites in primary hippocampal neurons, as indicated by the reduction of GluN2A co-localization with the postsynaptic marker Shank (Fig. 6c,d; **P=0.0069; unpaired Student's *t*-test). TAT-Rph3A-9c also reduced GluN2A surface staining (Supplementary Fig. 3i,j; *P=0.0130; unpaired Student's *t*-test) and GluN2A surface expression in PSD-95-positive postsynaptic clusters (Supplementary Fig. 3k,l; ***P=0.005; unpaired Student's *t*-test). Overall, these results indicate that Rph3A/PSD-95 interaction has a role in the surface stabilization of GluN2A at synaptic sites similar to Rph3A/GluN2A interaction.

### GluN2A/Rph3A/PSD95 complex modulates NMDA currents

**Figure 7:**
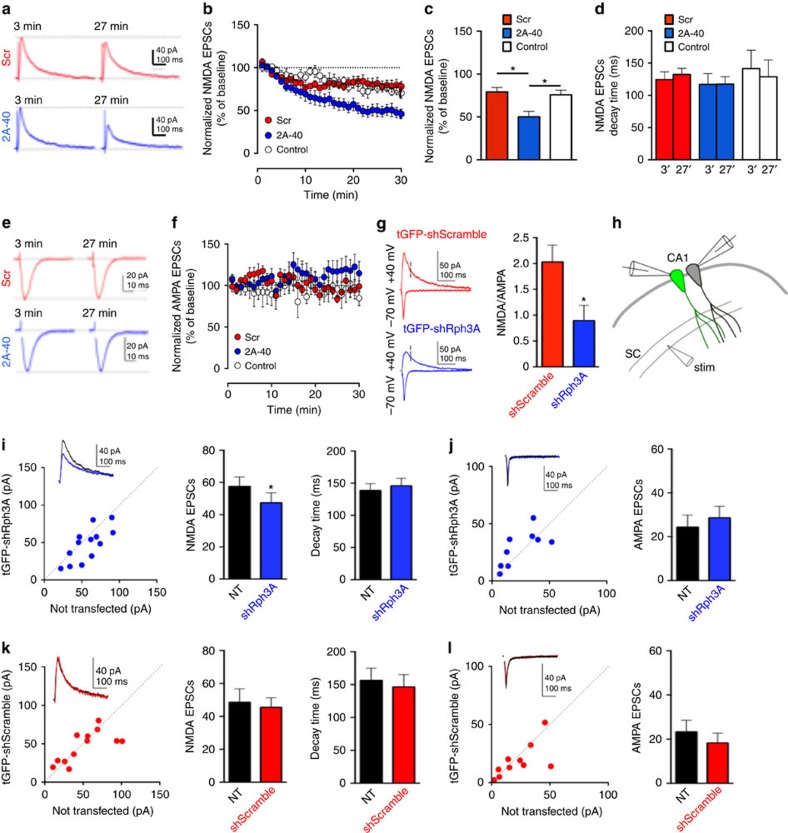
Effect of GluN2A/Rph3A interaction on NMDAR currents. (**a**) Sample traces showing the effect of intracellular perfusion of the non-permeable 2A-40 or its scramble peptides on pharmacologically isolated NMDAR-mediated EPSCs (+30 mV) recorded from a CA1 pyramidal cell. Traces represent the average of 6–9 responses with a scale of 40 pA over 100 ms. (**b**) Summary graph illustrating the time course of the effect of 2A-40 or its scramble on the peak amplitude of NMDAR-EPSCs. For comparison, the amplitude of NMDAR-EPSCs in the absence of any peptide in the intracellular solution (open circles) were also plotted (*n*=5–9). (**c**,**d**) Bar graphs summarizing the effect on the amplitude of NMDAR-EPSCs (**c**; Mann–Whitney test) and decay time (**d**) of the peptides (*n*=5–9). (**e**) Sample traces show the effect of intracellular perfusion of the non-permeable 2A-40 or its scramble control peptides on pharmacologically isolated AMPAR-mediated EPSCs (−70 mV). Traces represent the average of 6–9 responses with a scale of 20 pA over 10 ms. (**f**) Summary graph illustrating the time course of the effect of 2A-40 or its scramble on the peak amplitude of AMPAR-EPSCs. For comparison, it is also plotted (open circles) the amplitude of AMPAR-EPSCs in the absence of any peptide in the intracellular solution, *n*=6–7. (**g**) Sample traces and summary graphs illustrating that transfection of tGFP-shRNA-Rph3A induced a significant reduction in the NMDA/AMPA ratio when compared with cells transfected with tGFP-shRNA-Scramble (tGFP-shRNA-Scramble: 2.03±0.32; *n*=9; tGFP-shRNA-Rph3A: 0.89±0.29, *n*=8; Mann–Whitney test). (**h**) Schematic illustrating paired recordings from neighbouring transfected and non-transfected neurons. (**i**) Sample traces shown in the inset (not transfected (NT): *n*=13; tGFP-shRNA-Rph3A: *n*=13; Mann–Whitney test). (**j**) Amplitude of AMPAR-mediates EPSCs at SC-CA1 (recorded at −70 mV; NT: *n*=9; tGFP-shRNA-Rph3A: *n*=9). (**k**) Transfection with tGFP-shRNA-Scramble did not modify the amplitude or decay time of NMDAR-mediated EPSCs at SC-CA1 synapse (NT: *n*=12; tGFP-shRNA-Scramble: *n*=12). (**l**) Transfection with tGFP-shRNA-Scramble did not reduce the amplitude of AMPAR-mediated EPSCs at SC-CA1 (NT: *n*=10; tGFP-shRNA-Scramble: *n*=10). All data are represented as mean±s.e.m. *P<0.05.

Disruption of Rph3A/GluN2A/PSD-95 ternary complex decreases the levels of GluN2A at synapses. We directly tested whether the amplitude of synaptic NMDAR-currents was affected by performing patch-clamp recordings of pharmacologically isolated excitatory postsynaptic currents of NMDAR (NMDAR-EPSCs) in CA1 neurons from hippocampal organotypic slices (*DIV15*). We directly included a cell impermeable 2A-40 peptide (lacking the TAT sequence) into the patch-pipette intracellular solution enabling postsynaptic targeting of the peptide in a single cell. A scramble (Scr) peptide (also lacking TAT sequence) was used as a control. In the presence of the 2A-40 peptide (1 μM), the amplitude of NMDAR-EPSCs markedly decreased during 30 min of recording as compared with control conditions (Fig. 7a–c; *P<0.05 2A-40 versus Scr, *P<0.05 2A-40 versus control; Mann–Whitney test), with no effect on NMDAR-EPSC decay time (Fig. 7d; 2A-40 versus Scr). This effect was specific to NMDAR-EPSCs as no change was observed in the amplitude of AMPAR-EPSCs (Fig. 7e,f). In these experiments, we normalized the amplitude of the synaptic currents (both NMDAR and AMPAR mediated) with the average amplitude of the first 3 min of the experiment (see Methods for details). Therefore, disrupting Rph3A/GluN2A interaction selectively affects the amplitude of NMDAR-EPSCs, without modulating the kinetics of the synaptic response, consistent with a decrease of synaptic NMDARs.

To further confirm these observations, we tested the effect of knocking down Rph3A on glutamatergic synaptic transmission (Fig. 7g–l). We performed single-cell electroporation of CA1 pyramidal cells in organotypic hippocampal slices with either tGFP-shScramble or tGFP-shRph3A. Three to five days after transfection, we recorded the NMDA/AMPA ratio and found that the ratio was significantly reduced in neurons in which Rph3A has been knocked down (Fig. 7g, *P=0.0274; Mann–Whitney test). This result could be interpreted as either a decrease of NMDAR-mediated currents or an increase of AMPAR-mediated currents or both. To directly address this, we recorded pharmacologically isolated NMDAR-EPSCs from a transfected neuron and a neighbouring non-transfected neuron in pairs (Fig. 7h). Knock down of Rph3A significantly reduced the amplitude of synaptic NMDAR-mediated responses compared with control (Fig. 7i, *P=0.0370; Mann–Whitney test). The decay time of NMDAR-EPSCs did not vary between the two conditions, suggesting that the composition of synaptic NMDARs did not change (Fig. 7i). On the other hand, the amplitude of pharmacologically isolated AMPAR-EPSCs did not differ between cells transfected with shRph3A and non-transfected cells (Fig. 7j). Importantly, no significant difference in the amplitude and decay time of NMDAR-EPSCs or AMPAR-EPSCs was observed when cells were transfected with the tGFP-shScramble (Fig. 7k,l). Overall, these data confirm that the loss of Rph3A reduces the levels of synaptic NMDARs at hippocampal excitatory synapses.

### GluN2A/Rph3A/PSD95 complex modulates spine density

**Figure 8:**
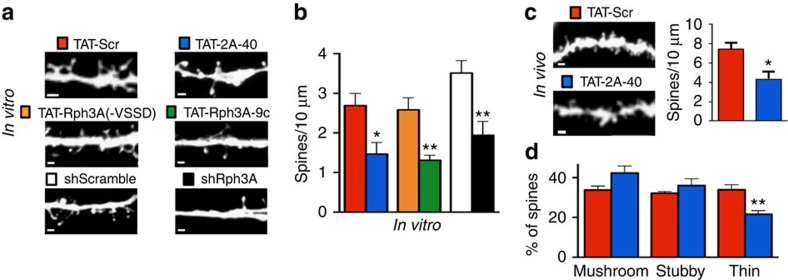
Effect of GluN2A/Rph3A interaction on spine morphology. (**a**) Representative images show dendrites of *DIV15* hippocampal neurons treated with TAT-Scr (red), TAT-2A-40 (blue), TAT-Rph3A(−VSSD; orange) or TAT-Rph3A-9c (green) or transfected with tGFP-shScramble (white) or tGFP-shRph3A (black) at *DIV9*. (**b**) Bar graph representing the respective spine densities in these neurons (*n*=6–8; unpaired Student's *t*-test). (**c**) Representative images show dendrites of adult mouse CA1 neurons 2 h after injection of either TAT-Scr (red) or TAT-2A-40 (blue), both 3 nmol g^−1^ i.p. Bar graph represents the respective spine densities in these neurons (*n*=5; unpaired Student's *t*-test). (**d**) Bar graph representing the different spine types percentage compared with the total spines (mushroom, stubby and thin; *n*=5; unpaired Student's *t*-test). All data are presented as mean±s.e.m. *P<0.05, **P<0.01, ***P<0.001. Scale bars, 1 μm.

The role of GluN2 subunits in dendritic spine remodelling has not been fully investigated^8^,^36^,^37^. We evaluated the role of the postsynaptic Rph3A/GluN2A/PSD-95 complex on dendritic spine morphology by acute treatment (1 h) of primary hippocampal neurons with TAT-2A-40 or TAT-Rph3A-9c (and corresponding control peptides) and by knock-down of Rph3A with tGFP-shRph3A. All experimental approaches led to a significant reduction in dendritic spine density compared with control conditions (Fig. 8a,b; *P=0.0165, TAT-2A-40 versus TAT-Scr; **P=0.0019, TAT-Rph3A-9c versus TAT-Rph3A(−VSSD); **P=0.0053, tGFP-shRph3A versus tGFP-shScramble; unpaired Student's *t*-test). Accordingly, we also observed a reduction in PSD-95-positive clusters density in neurons (*DIV15*) transfected with tGFP-shRph3A compared with tGFP-shScramble controls (Supplementary Fig. 4a,b, **P<0.01; unpaired Student's *t*-test). We also found that Rph3A silencing in primary hippocampal cultures induced a mild but not significant (*P*=0.055; unpaired Student's *t*-test) decrease in PSD-95 expression in total neuronal lysates (*DIV15*; Supplementary Fig. 4c,d). For a more detailed morphological analysis, the dendritic spine length, head and neck width were measured and then, spines were categorized according to their shape (mushroom, stubby, thin and filopodia) using a highly validated classification method^38^. No statistically significant difference in spine type was observed following *in vitro* treatment with CPPs and Rph3A shRNA (Supplementary Fig. 4e–g). In addition, the above-described treatments did not modify the percentage of spine versus filopodia-like protrusions (Supplementary Fig. 4h–j). *In vivo* acute treatment (2 h before sacrifice) of mice with TAT-2A-40 (3 nmol g^−1^, i.p.) led to a similar decrease in spine density in CA1 hippocampal neurons (Fig. 8c, *P=0.0184; unpaired Student's *t*-test) and also induced a significant modification of spine type, namely a reduction of the percentage of thin spines (Fig. 8d; **P=0.0037; unpaired Student's *t*-test).

### Disruption of the Rph3A/GluN2A/PSD-95 complex *in vivo*

At birth, the abundance of GluN2A-containing NMDARs is very low in the hippocampus^7^, their expression increases during development, starting from the second post-natal week^8^. This accumulation of GluN2A-containing NMDARs is crucial for the maturation of excitatory synapses^7^. We observed that Rph3A expression in the hippocampus increased during development in parallel to GluN2A (Supplementary Fig. 5a). Because of the role of the Rph3A/GluN2A interaction in stabilizing GluN2A at synapses, we tested whether this interaction was important for the developmental increase of synaptic GluN2A.

**Figure 9:**
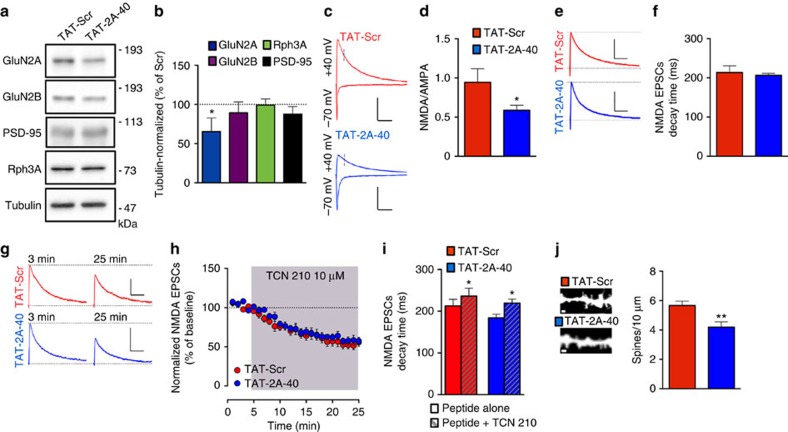
Modulation of GluN2A-containing NMDAR expression at synapses in the developing rat hippocampus. (**a**) Western blot analysis of GluN2A, GluN2B, PSD-95, Rph3A and tubulin of TIF from treated rat pups hippocampus. (**b**) Bar graph representing the percentage of tubulin normalized integrated density of GluN2A, GluN2B, Rph3A and PSD-95 WB bands from TIF samples compared with their respective TIF purification TAT-Scr control (*n*=3–5; unpaired Student's *t*-test). (**c**) Sample traces illustrating a decreased NMDA/AMPA in TAT-2A-40-treated animals (blue) compared with TAT-Scr-treated (red) animals at Schaffer collaterals to CA1 pyramidal cell synapses in acute hippocampal slices with 100 pA over 100 ms scale. (**d**) Bar graph summarizes the significant decrease in NMDA/AMPA in TAT-2A-40-treated animals (blue) compared with TAT-Scr-treated (red) animals (*n*=9–12; Mann–Whitney test). (**e**,**f**) Sample traces (**e**) and summary bar graph (**f**) illustrating that the decay time of pharmacologically isolated NMDAR-EPSCs does not differ between TAT-Scr-treated (red) and TAT-2A-40-treated (blue) animals (*n*=10–14) with 40 pA over 100 ms scale. (**g**–**i**) Sample traces (**g**) and summary graphs illustrating that the amplitude (**h**) and decay time (**i**) of the pharmacologically isolated NMDAR-EPSCs are equally modulated by application of TCN 210 in both conditions (*n*=6–7; Wilcoxon test) with 50 pA over 200 ms scale. (**j**) Representative images show dendrites of P15 rat pups CA1 neuron either treated with TAT-Scr (red) or TAT-2A-40 (blue), both 3 nmol g^−1^ s.c. chronically from P6 to P14. Bar graph represents the respective spine densities (*n*=10–11; unpaired Student's *t* test). All data are presented as mean±s.e.m. *P<0.05, **P<0.01, ***P<0.001. Scale bars, 1 μm.

We chronically treated rat pups with TAT-2A-40 or its control TAT-Scr peptide (5 injections every other day, starting at P6 and ending at P14, 3 nmol g^−1^ subcutaneous (s.c.)). The animals were then killed at P15 for molecular, electrophysiological and spine morphology analyses. Biochemical analysis showed that the chronic treatment with TAT-2A-40 led to a significant decrease of GluN2A enrichment in a Triton X-100-insoluble postsynaptic fraction (TIF; Fig. 9a,b; *P=0.0340 as compared with TAT-Scr; unpaired Student's *t*-test) with no changes in Rph3A, GluN2B and PSD-95 synaptic levels (Fig. 9a,b).

We next assessed the functional consequences of chronic disruption of Rph3A/GluN2A interaction on synaptic transmission in patch clamp recordings from CA1 pyramidal cells in acute slices. We found no difference in the amplitude and frequency of spontaneous synaptic events (miniature excitatory postsynaptic current (mEPSC)), in tetrodotoxin and recorded at −70 mV; Supplementary Fig. 5b–d), or in the paired pulse ratio (40 ms and recorded at −70 mV; Supplementary Fig. 5e,f). These data indicate that chronic disruption of the Rph3A/GluN2A complex does not affect the amount of synaptic AMPARs (mEPSC amplitude) and probability of release (mEPSC frequency and paired pulse ratio), further confirming the postsynaptic locus of action of the TAT-2A-40 peptide. We then estimated the changes in synaptic NMDARs by measuring synaptic NMDA/AMPA ratios knowing that the content of synaptic AMPAR was not affected following TAT-2A-40 treatment. AMPAR-EPSCs and NMDAR-EPSCs were recorded from CA1 pyramidal cells, respectively, at −70 and +40 mV. The NMDAR-mediated contribution was measured 50 ms after initiation of the EPSC, a time point at which the contribution of AMPAR-EPSCs is absent or minimal. Treatment with TAT-2A-40 induced a significant decrease in the NMDA/AMPA ratio compared with TAT-Scr treatment (Fig. 9c,d, *P=0.0169; Mann–Whitney test).

Chronic treatment with TAT-2A-40 induced a significant decrease in the amount of GluN2A but not GluN2B in synaptic fractions (Fig. 9a,b). Decrease in the relative abundance of GluN2A with respect to GluN2B could result in the slowing down of the kinetics of NMDAR-EPSCs^6^. Rather unexpectedly, the kinetics of pharmacologically isolated NMDAR-EPSCs (in the presence of NBQX 20 μM and recorded at +40 mV) were not different among the various treatments (Fig. 9e,f). To confirm this result, we tested the effect of TCN-201 (10 μM), a selective blocker of GluN2A-containing NMDARs^39^, on pharmacologically isolated NMDAR-EPSCs. We found that amplitude and kinetics of NMDAR-EPSCs were similarly inhibited by this drug (Fig. 9g–i; amplitude: *P=0.0313; decay time: *P=0.0313; Wilcoxon test).

Moreover, chronic treatment of young rats with TAT-2A-40 induced a decrease in spine density of CA1 hippocampal neurons (Fig. 9j; **P=0.0055; unpaired Student's *t*-test), as following acute treatments (see Fig. 8 for details), without any significant difference in the proportion of different spine types (Supplementary Fig. 5g). In agreement with the effect on spine density, we found a decrease in PSD-95 expression in total homogenates in the hippocampus of TAT-2A-40 chronically treated pups compared with their respective TAT-Scr controls (Supplementary Fig. 5h; **P=0.0026; unpaired Student's *t*-test). Interestingly, two other administration protocols, (i) animals treated by a single injection of the CPPs at 3 nmol g^−1^ s.c. at P6 and killed at P15 and (ii) animals treated by a chronic injection of the CPPs starting at P6 and ending at P14 and killed at P21, led to a similar decrease of GluN2A-containing NMDARs at synaptic sites (Supplementary Fig. 5i,j; 1: *P=0.0212, 2: *P=0.023; unpaired Student's *t*-test), thus indicating the persistency of the molecular effect induced by the disruption of GluN2A/Rph3A complexes.

## Discussion

GluN2A-containing NMDARs are known to be less mobile and more stable at synapses, as compared with GluN2B-containing NMDARs^1^,^2^,^7^. However, data on the detailed molecular mechanisms responsible for the synaptic retention of GluN2A-containing NMDARs are currently insufficient. Our results indicate that Rph3A, previously thought to be purely presynaptic, is required for the stabilization of GluN2A-containing NMDARs at the postsynaptic membrane in hippocampal neurons, through the formation of a ternary GluN2A/Rph3A/PSD-95 complex.

At the presynaptic level^20^, Rph3A modulates synaptic vesicle trafficking^21^. Here we show that Rph3A is not localized exclusively in presynaptic compartments, but is also present in the postsynaptic compartment. In particular, electron microscopy studies indicate the presence of Rph3A at dendritic spines and at the edge of the excitatory PSD. Notably, our results are in agreement with several proteomic studies all converging on the identification of Rph3A as a member of the PSD complex^40^,^41^,^42^,^43^. In addition, previous studies indicate that Rph3A can interact with postsynaptic proteins^24^,^44^,^45^, some of these (α-actinin, Myosin-Va) being able to associate directly to ionotropic glutamate receptors^45^,^46^. Finally, the Rph3A interaction with PSD-95 described here is consistent with the previously shown capability of Rph3A to bind CASK, another member of the MAGUKs family of scaffolding proteins^22^.

In line with the postsynaptic localization, we show that Rph3A specifically and directly interacts with GluN2A and with PSD-95. Our data indicate that Rph3A forms a ternary complex with GluN2A and PSD-95. Notably, disruption of either GluN2A/Rph3A or PSD-95/Rph3A interactions leads to a decreased stability of GluN2A/PSD-95 protein interaction inducing a rapid decrease of GluN2A-containing NMDARs inserted in the postsynaptic membrane. Time-lapse confocal imaging experiments with SEP-GluN2A and measurement of NMDAR currents concur with a reduction of active NMDARs at the cell surface within 10–15 min after disruption of GluN2A/Rph3A complex.

Previous reports indicated the presence of stable endocytic zones on the spine surface near the PSD^47^,^48^,^49^,^50^. Notably, here we show by electron microscopy studies that Rph3A is localized at the postsynaptic membranes in close proximity of the PSDs thus in a strategic position to interfere with receptor endocytosis through stabilization of the GluN2A/PSD-95 complex. In agreement with this hypothesis, blocking receptor endocytosis with the dynamin-inhibitor dynasore^35^ fully prevents GluN2A reduction at the cell surface induced by GluN2A/Rph3A complex disruption. Hence, Rph3A stabilizes GluN2A in the synaptic membrane and prevents endocytosis likely by strengthening synaptic GluN2A/PSD-95 interaction.

It is well-known that levels and expression patterns of the GluN2 subunits change during the first two postnatal weeks. In particular, GluN2A expression increases from the second postnatal week to become widely expressed and abundant throughout the brain^8^,^51^. Notably, several studies have demonstrated that GluN1/GluN2A/GluN2B tri-heteromers account for at least two-thirds of the total NMDARs in the adult hippocampus, thus indicating that NMDARs clustering, synaptic targeting and function at hippocampal synapses are mainly determined by these tri-heteromeric receptors^8^,^51^,^52^. In agreement with this idea, we show that a peptide perturbing Rph3A interaction with GluN2A induces a selective decrease in the overall amplitude of NMDAR-EPSCs, without modulating the kinetics of the synaptic response, suggesting that there are fewer GluN1/GluN2A/GluN2B receptors available for synaptic activation. Interestingly, both Rph3A shRNA and GluN2A/Rph3A interfering peptide in hippocampal organotypic slices (DIV15) and chronic *in vivo* systemic administration of the peptide from P6 to P14 rats led to the decrease in NMDAR-EPSCs.

GluN2A/Rph3A interfering peptides and Rph3A silencing also caused a significant decrease in dendritic spine density with no modification of size and overall morphology. A possible correlation between GluN2A-containing NMDARs and dendritic spine morphology has been previously proposed. In particular, in agreement with our results, IQGAP1-dependent impairment of surface expression of GluN2A-containing NMDARs in hippocampal neurons led to a significant reduction of spine density^37^. Interestingly, no significant alterations in dendritic spines number were observed in experimental conditions of more direct and dramatic reduction of GluN2A levels^8^,^36^. This suggests that a fine modulation of GluN2A surface synaptic localization but not a more general subunit knock-down could cause changes in synaptic morphology.

Several studies have proposed that abnormal GluN2-type subunits trafficking, resulting in the modification of the NMDAR subunit composition at synapses, has a major role in the pathogenesis of several brain disorders^1^,^53^,^54^. Moreover, recent observations clearly indicate that alterations in PSD-MAGUK protein interaction with GluN2 subunits are common events in several neurodegenerative disorders^10^. Interestingly, specific reductions in Rph3A immunoreactivity in neurodegenerative disorders, such as Alzheimer's disease^55^, α-synucleinopathy^56^ and Huntington's disease^57^ were observed. Accordingly, Rph3A with its specific role in modulating synaptic GluN2A-containing NMDARs could represent an innovative target to rescue an altered NMDAR subunit composition in CNS disorders.

**Figure 10:**
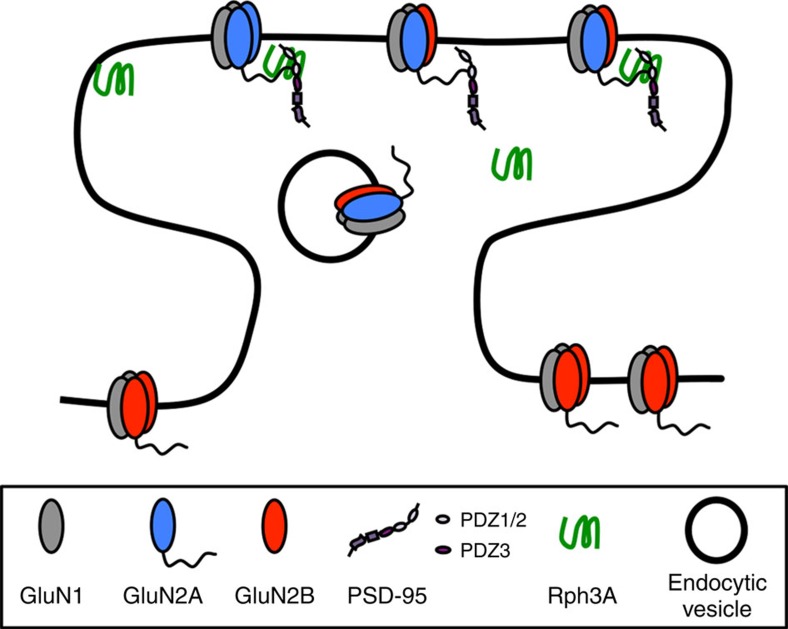
Schematic of GluN2A/Rph3A/PSD-95 ternary complex at the PSD. Rph3A is involved in a ternary complex with GluN2A and PSD-95 to help stabilize GluN2A-containing NMDARs at the synaptic membrane. Rph3A localized at the lateral domain of the PSD interacts with the cytoplasmic C-terminal tail of GluN2A and the PDZ3 domain of PSD-95, whereas GluN2A can bind to PDZ1 or PDZ2 of PSD-95. Disruption of one of these interactions is enough to reduce the amount of GluN2A-containing NMDARs at synaptic membranes affecting the overall amplitude of NMDARs response and spine density in the hippocampus.

Altogether, here we report that Rph3A takes part in GluN2A/PSD-95 complexes playing a crucial role in synaptic retention of GluN2A-containing NMDARs (Fig. 10).

## Methods

### Animals

C57/BL6 mice of 6 weeks, Sprague–Dawley rats of 6 weeks, E18 embryos from Sprague–Dawley rats for primary hippocampal neuron cultures and P6-P21 Sprague–Dawley rat pups were used. All the experiments were approved by the OHSU Institutional Animal Care and Use Committee and by the Italian Health Ministry (#295/2012-A). Likewise, all animals were used according to the guidelines of the University of Bordeaux/CNRS Animal Care and Use Committee and the requirements of the United Kingdom Animals (Scientific Procedures) Act 1986.

### Y2H screening

Y2H was conducted according to the manufacture's procedure guidelines using the Mate & Plate Library—Mouse Brain (normalized; cat #630488, TakaraBio/Clontech Europe, France). Briefly, the GluN2A(839–1461) C-tail (bait) was cloned in the pGBKT7 plasmid and transformed in the AH109 haploid yeast strain (MATα). This was mated overnight with the Mouse Brain normalized library (prays) cloned in pGADT7 transformed in Y187 haploid yeast strain (MATα). Yeast were plated after 24 h on selective plate, allowing only the growth of diploid where a protein interaction between the bait and pray protein occurred (absence of Leucine, Adenine, Tryptophan and Histidine). Then the diploids were tested by a colorimetric assay (α-gal) to avoid the presence of false positive. Plasmids (twenty-one positive clones) were extracted from the yeast and sequenced. Five genes were identified following sequencing, one of those being Rph3A.

### Antibodies

The following primary antibodies were used: rabbit anti-N-term GluN2A (480031, Invitrogen; dilution 1:100 ICC), rabbit anti-GluN2A (M264, Sigma-Aldrich; dilution 1:1,000 WB), mouse anti-N-term GluN2B (75–101, Neuromab; dilution 1:100 ICC, 1:1,000 WB), anti-PSD-95 (75–028; Neuromab; dilution 1:250 ICC, 1:1,000 WB) and anti-Pan-shank (75–089, Neuromab; dilution 1:200), rabbit anti-N-term GluA1 (MAB2263, Millipore; Billenca, MA; dilution 1:100 ICC), rabbit anti-Rph3A (NB100–92221, Novus biological; dilution 1:100 ICC), mouse anti-Rph3A (RM3701, ECM biosciences; dilution 1:100 ICC, 1:1,000 WB), rabbit anti-Rab8 (D22D8, Cell Signaling; dilution 1:1,000 WB), mouse anti-Meox2 (ab117551, Abcam; dilution 1:1,000 WB), mouse anti-Tubulin (T9026, Sigma-Aldrich; dilution 1:5,000 WB). The following secondary antibodies were used: goat anti-mouse-HRP (172–1,011) and goat anti-rabbit-HRP (172–1,019, Bio-Rad), goat anti-mouse-Alexa488 (A-11029), goat anti-mouse-Alexa555 (A-21422), goat anti-mouse-Alexa633 (A-21052), goat anti-rabbit-Alexa488 (A-11034) and goat anti-rabbit-Alexa555 (A-21429; Life Technologies). For the original WB, please see Supplementary Fig. 6.

### Plasmids

RFP-Rph3A plasmid was a kind gift from Professor Mitsunori Fukuda (University of Tokyo, Meguro, Tokyo, Japan). This plasmid was used to perform point mutation inserting a stop codon for the codon corresponding to aa 673, 380 or 179. PSD-95 fusion proteins plasmids were provided by Professor Yutaka Hata (Tokyo Medical and Dental University, Japan). eGFP-GluN2A was provided by Professor Richard Huganir (Johns Hopkins University School of Medicine, Baltimore, MD, USA). This plasmid was used to perform point mutation inserting a stop codon for the codon corresponding to aa 1,049. eGFP was provided by Dr Maria Passafaro (CNR, Milan, Italy). SEP-GluN2A and SEP-GluN2B were provided by Professor Roberto Malinow through Addgene as plasmid #23997 and plasmid #23998, respectively^58^.

### Cell-permeable peptides

Each CPP was manufactured by Bachem (Bubendorf, Switzerland) according to our designed sequences as follows. TAT-Rph3A-9c: YGRKKRRQRRR-QNENHVSSD; TAT-Rph3A(−VSSD): YGRKKRRQRRR-QNENH; TAT-2A-40: YGRKKRRQRRR-EDSKRSKSLLPDHASDNPFLHTYQDDQRLVIGRCDSDPYKH; TAT-Scr: YGRKKRRQRRR-LDPHSNPLYCPDLYSERFDVSKDHRLDKTKSDHAQDRASIG. Lyophilized CPPs were resuspended in sterile deionized water to a stock concentration of 1 mM and stored at −20 °C. *In vitro* treatments were performed with a 1–10 μM concentrations of CPPs, *in vivo* treatments were performed by injecting CPPs at 3 nmol g^−1^.

### Cell cultures and transfections

COS7 cells were grown on 100 mm dishes and maintained in DMEM containing Glutamax (DMEM+Glutamax, GIBCO) supplemented with 10% fetal bovine serum and penicillin–streptomycin (GIBCO). Cells were allowed to grow till confluence before passaging every 3–4 days using trypsin. The day before transfection, COS-7 cells were placed in a 12 wells multiwell (for imaging), then cells were transfected with 250–500 ng of plasmid DNA (RFP-Rph3A; RFP-Rph3A(673); eGFP-GluN2A; eGFP-GluN2A(1,049); PSD-95) using the lipofectamine LTX method (invitrogen). After 36 h, COS-7 cells were fixed for immunostaining/imaging. Hippocampal neuronal primary cultures were prepared from embryonic day 18–19 (E18-E19) rat hippocampi^59^. Neurons were transfected at *DIV9* using calcium-phosphate co-precipitation method with 2–4 μg of plasmid DNA for: RFP-Rph3A; eGFP-GluN2A; eGFP; shRph3A-tGFP or shScramble-tGFP, (Thermoscientific). Neurons were treated at *DIV15*, fixed and then immunostained.

### Fluorescent immunocytochemistry

Cells were fixed with 4% Paraformaldehyde (PFA)-4% sucrose in PBS solution at 4 °C and washed several times with PBS. Cells were either blocked with 5% BSA in PBS for 30 min at room temperature and then labelled with primary antibodies for surface labelling for 1 h at room temperature or permeabilized with 0.1% Triton X-100 in PBS for 15 min at room temperature and then blocked with 5% BSA in PBS for 30 min at room temperature. Cells were then labelled with antibodies for intracellular epitopes for 1 h at room temperature or overnight at 4 °C. Cells were washed and then incubated with secondary antibodies for 1 h at room temperature. Cells were then washed in PBS and mounted on glass slides with Fluoromount mounting medium (Sigma Aldrich) or permeabilized for total labelling.

### Pre-embedding immunocytochemistry

Three Sprague–Dawley male rats (∼6-week-old) of ∼150 g were used. After terminal anaesthesia was induced by brief inhalation of isoflurane (0.05% in air), followed by an intramuscular injection of ketamine (100 mg kg^−1^) and xylazine (10 mg kg^−1^), rats were intracardially perfused with 4% PFA and 0.1% glutaraldehyde in PBS (0.1 M, pH 7.2), and brain sections (100 μm) were cut on a Leica VT1000S vibratome (Leica Microsystems)^19^,^60^. The sections were immunolabelled with rabbit anti-Rph3a polyclonal antibody (1:100; ab68857, Abcam), followed by a biotinylated secondary antibody, ABC Elite Kit (Vector Laboratories), and the peroxidase reaction was revealed by ImmPACT VIP substrate Kit (Vector Laboratories). Then the sections were osmicated, dehydrated and flat embedded in Durcupan resin (Sigma-Aldrich). Ultrathin sections (70–90 nm) were countercoloured with uranyl acetate and lead citrate. Control experiments, in which the primary antibody was omitted, resulted in no immunoreactivity. The sections were visualized with a Philips CM100 transmission electron microscope (FEI) at 100 kV. The images were captured with an AMT XR40 4 megapixel side mounted CCD camera at a magnification between × 7,900 and × 92,000.

### Computational procedures

All the computational procedures were carried out through the Molecular Operating Environment (MOE) version 2014.0901 from the Chemical Computing Group. The Amber99 forcefield^61^ with the Generalized Born implicit solvation method^62^ was used for all the computational tasks.

The crystallographic structure of the PDZ3 domain of rat PSD-95 co-crystallized with a C-terminal peptide derived from the CRIPT protein (PDB ID: 1BE9)^34^ was downloaded and submitted to the Structure Preparation procedure. The binding free energy between PSD-95 and the CRIPT peptide was computed through the MOE LigX procedures, useful to minimize the crystallographic structure and to estimate the binding affinity through the forcefield-based GBVI/WSA dG scoring function, which estimates the free energy of interaction. The complex between the PSD-95 PDZ3 domain and the C-terminus of Rph3A was obtained by mutating the CRIPT peptide into HVSSD primary structure with the Protein Design module. The binding free energy was computed with the MOE LigX as above.

### Subcellular fractionations

TIF, a fraction highly enriched in PSD proteins but absent of presynaptic markers^63^, was isolated from adult rat hippocampus. To this, samples were homogenized at 4 °C in an ice-cold buffer containing 0.32 M Sucrose, 1 mM HEPES, 1 mM NaF, 0.1 mM phenylmethylsulfonyl fluoride (PMSF), 1 mM MgCl in the presence of protease inhibitors (Complete, GE Healthcare) and phosphatase inhibitors (PhosSTOP, Roche Diagnostics GmbH), using a glass-Teflon homogenizer. Homogenates were then centrifuged at 1,000*g* for 5 min at 4 °C, to remove nuclear contamination and white matter. The supernatant was collected and centrifuged at 13,000*g* for 15 min at 4 °C. The resulting pellet (P2 crude membrane fraction) was resuspended in hypotonic buffer (1 mM HEPES with Complete). Resuspended P2 were then centrifuged at 100,000*g* for 1 h at 4 °C. Triton X-100 extraction of the resulting pellet was carried out at 4 °C for 20 min in an extraction buffer (1% Triton X-100, 75 mM KCl and Complete). After extraction, the samples were centrifuged at 100,000*g* for 1 h at 4 °C and the TIFs obtained were resuspended in 20 mM HEPES with Complete. PSDs were isolated from rat hippocampus^64^. Rats (15 animals) were killed, hippocampi were dissected within 2 min and pooled. All hippocampi dissected in >2 min were discarded. Homogenization was carried out by 10 strokes in a glass-Teflon homogenizer (700 r.p.m.) in 4 ml g^−1^ of cold 0.32 M sucrose containing 1 mM HEPES, 1 mM MgCl_2_, 1 mM NaHCO_2_ and 0.1 mM PMSF (pH 7.4). The homogenized tissue was centrifuged at 1,000*g* for 10 min. The resulting supernatant was centrifuged at 13,000*g* for 15 min to obtain a fraction containing mitochondria and synaptosomes. The pellet was resuspended in 2.4 ml g^−1^ of 0.32 M sucrose containing 1 mM HEPES, 1 mM NaHCO_3_ and 0.1 mM PMSF, overlaid on a sucrose gradient (0.85–1.0–1.2 M), and centrifuged at 82,500*g* for 2 h. The fraction between 1.0 and 1.2 M sucrose was removed, diluted with an equal volume of 1% Triton X-100 in 0.32 M sucrose containing 1 mM HEPES, 15 min. This solution was spun down at 82,500*g* for 45 min. The pellet (Triton insoluble postsynaptic fraction, PSD1) was resuspended, layered on a sucrose gradient (1.0–1.5–2.1 M), and centrifuged at 100,000*g* at 4 °C for 2 h. The fraction between 1.5 and 2.1 M was removed and diluted with an equal volume of 1% Triton X-100 and 150 mM KCl. PSD2 were finally collected by centrifugation at 100,000*g* at 4 °C for 45 min and stored at −80 °C until processing. All purifications were performed in the presence of complete sets of protease and phosphatase inhibitors (Roche Diagnostics). Protein content of the samples was quantified by using Bio-Rad protein assay. After measuring protein concentration, all samples were standardized at 1 μg μl^−1^ concentration and the same protein amount loaded in each lane. For the original WB, please see Supplementary Fig. 6.

### Co-IP assay

A measure of 50 μg of proteins from rat hippocampus or mice forebrain P2 fractions were incubated for 1 h at 4 °C in RIA buffer containing 200 mM NaCl, 10 mM EDTA, 10 mM Na_2_HPO_4_, 0.5% NP-40, 0.1% SDS and protein A/G-agarose beads as pre-cleaning procedure. The beads were then let to sediment at the bottom of the tube and the supernatant was collected. Primary antibodies were added to the supernatant before leaving to incubate overnight at 4 °C on a wheel. Protein A/G-agarose beads were added and incubation was continued for 2 h, at room temperature on a wheel. Beads were collected by gravity and washed three times with RIA buffer before adding sample buffer for SDS–polyacrylamide gel electrophoresis (SDS–PAGE) and the mixture was boiled for 10 min. Beads were pelleted by centrifugation, all supernatants were applied onto 7–12% SDS–PAGE gels. For the original WB, please see Supplementary Fig. 6.

### Pull-down assay

GST fusion proteins were expressed in *Escherichia coli* and purified on glutathione agarose beads (Sigma Aldrich)^26^. A measure of 250 μg of brain homogenate proteins were incubated with 20 μl of GST alone proteins to a final volume of 1 ml in Tris-Buffered Saline (TBS, 10 mM Tris and 150 mM NaCl) for 1 h on the rotator at room temperature. Samples were then centrifuged at 3,000 r.p.m. for 90 s and the supernatants were incubated for 2 h with 40 μl of GST fusion proteins of the C-terminal domains of GluN2A, PDZ1–2 or PZ3 domains of PSD-95 or GST alone. After incubation, beads were washed four times with TBS and 0.1% Triton X-100. For the Ca^2+^/IP_3_ experiments, 175 μg of hippocampal homogenate proteins were incubated with 10 μl of GST alone proteins to a final volume of 500 μl of TBS for 1 h on the rotator at room temperature. Samples were then centrifuged at 3,000 r.p.m. for 90 s and the supernatants were incubated for 2 h at room temperature with 25 μl of GST-GluN2A(1,049–1,464) or GST alone fusion proteins along with 20 mM Ca^2+^, 1 mM IP_3_ or 20 mM Ca^2+^ and 1 mM IP_3_. After incubation, beads were washed four times with TBS and 0.1% Triton X-100. Bound proteins were resolved by SDS–PAGE and subjected to immunoblot analysis. For the original WB, please see Supplementary Fig. 6.

### Slice electrophysiology

Organotypic hippocampal slice cultures were prepared from P5–P7 rat pups^65^, according to guidelines of the University of Bordeaux/CNRS Animal Care and Use Committee. Three to four days after plating, the medium was replaced and then changed every 2–3 days. Electrophysiological experiments were performed after 10–16 days in culture. After 7–9 days *in vitro,* we performed single-cell electroporation to transfect individual cells with cDNAs encoding tGFP-shRNA-Scramble or tGFP-shRNA-Rph3A. Plasmids (50 ng/μl^−1^) were dissolved in a filtered K+-based intracellular solution to fill 6–9 MΩ patch clamp pipettes. Electroporation was performed in a pre-warmed HEPES-based artificial cerebrospinal fluid (ACSF) by the delivery of 50–100 square-pulses (600 μs duration) at 100 Hz with an amplitude of −10 mV. Electrophysiological recording were performed 3–5 days after transfection.

For acute slices recordings, sagittal hippocampal slices (330 μm thick) were obtained from 15-day-old rat killed by cervical dislocation, 24 h after the last i.p. injection either with TAT-2A-40 or its scramble version (4 animals per condition were used). Acute slices were prepared using sucrose-based cutting solution and used until a maximum of 6 h after cutting. Slices were transferred to a recording chamber in which they were continuously superfused with an oxygenated extracellular solution (95% O_2_ and 5% CO_2_) containing 125 mM NaCl, 2.5 mM KCl, 1.25 mM NaH_2_PO_4_, 26 mM NaHCO_3_, 2.3 mM CaCl_2_, 1.3 mM MgCl_2_ and 25 mM glucose.

Whole-cell voltage-clamp recordings (3.5–4.5 Mω electrodes) were made from CA1 pyramidal cells visualized by infrared videomicroscopy. Slices were perfused with an extracellular solution identical to the one used for slice incubation. Bicuculline (10 μM) was added to the bath to block GABA_A_ receptors. The intracellular solution was composed of: 140 mM cesium methanesulfonate, 2 mM MgCl_2_, 4 mM NaCl, 5 mM phosphocreatine, 2 mM Na2ATP, 10 mM EGTA, 10 mM HEPES, 0.33 mM GTP (pH 7.3). Experiments in slice cultures were performed in the presence of N^6^-cyclopentyladenosine (50 nM) to reduce polysynaptic activity. Evoked postsynaptic responses were induced in CA1 pyramidal cells by stimulating Schaffer collaterals in stratum radiatum (0.1 Hz) with a monopolar glass pipette. After obtaining the cell attached configuration, we waited 5–10 min before entering in whole cell while stimulating Schaffer collateral with an intensity just below the threshold required to elicit an action potential monitored in cell attached configuration^6^. After entering whole cell, we waited 1–3 min before starting the recording to adjust the stimulation intensity to have similar staring amplitude (between 100 and 200 pA) of the NMDAR-EPSCs in all the experimental conditions. AMPAR-mediated EPSCs were recorded at −70 mV in the presence of competitive NMDAR blocker AP5. In experiments shown in Fig. 7a–f, nonpermeable 2A-40 or its scramble peptides (both at 1 μM) were delivered postsynaptically through the recording pipette; ‘Control' refers to experiments in which the intracellular solution did not contain any peptides. In experiments in which the peptides were dialysed via the patch pipette, we normalized the amplitude of the synaptic currents (both NMDA and AMPA receptor mediated) with the average amplitude of the first 3 min of the experiment. Statistical comparison was done between the average of the synaptic currents of the first 3 min with respect to the amplitude of the between minutes 27th and 30th.

Pharmacologically isolated NMDAR-mediated EPSCs were recorded at +30 mV (Fig. 7a–d) and +40 mV (Figs 7g,i,k and 9). NMDAR-mediated EPSCs decay time was calculated with a single weighted decay measure (referred in the text as decay time) and calculated from the area under the peak-normalized current for 0.7 s after the peak. For AMPA/NMDA, NMDAR-mediated EPSCs were measured 50 ms after the initiation of the EPSC, a time point at which AMPAR-mediated currents are absent or minimal. Small, hyperpolarizing voltage/current steps were given before each afferent stimulus allowing on-line monitoring of input and series resistance. The access resistance was <20 MW, and cells were discarded if it changed by >20%. No series resistance compensation was used. Recordings were made using an EPC 10 amplifier (HEKA Elektronik) and were filtered at 0.5–1 kHz, digitized at 5 kHz and stored on a personal computer. Analysis was performed using Neuromatic (www.neuromatic.thinkrandom. com) written within the Igor Pro 6.0 environment (WaveMetrics). Values are presented as mean±s.e.m.

### Spine morphology

Carbocyanine dye DiI (Invitrogen) was used to label neurons^66^. DiI crystals were applied using a thin needle by delicately touching region of interest on both sides of 2 mm coronal sections prepared from previously cardiacally fixed with 1.5% PFA in 0.1 M phosphate buffer (PB). DiI was left to diffuse for 1 day in the dark at room temperature, then sections were fixed again with 4% PFA in PB 0.1 M for 45 min at 4 °C. 150 μm coronal sections were then obtained using a vibratome, the first section was discarded. Sections were mounted on glass slides with Fluoromount mounting medium (Sigma-Aldrich) for confocal imaging. DiI crystals were also used on primary hippocampal neurons previously fixed with 1.5% PFA-4% sucrose in 0.1 M PB, where they were applied to the coverslip with a thin needle and let to diffuse for 30 min in 0.1 M PB and then fixed with 4% PFA-4% sucrose in PB for 5 min at 4 °C. After washing, coverslips were mounted on glass slides with Fluoromount mounting medium (Sigma-Aldrich) for confocal imaging.

### Confocal imaging

Images were taken using an inverted LSM510 confocal microscope (Zeiss) and analysed using either the AIM 4.2 software (Zeiss) for co-localization analysis or ImageJ software. SEP-GluN2A/dTom or SEP-GluN2B/dTom were imaged on an inverted LSM510 confocal microscope (Zeiss) in B27-supplemented neurobasal medium without phenol red. To test the population of surface SEP-GluN2A/B (provided by Professor Roberto Malinow through Addgene as plasmid #23997 and plasmid #23998, respectively)^58^, we used low pH-solution adjusted to pH 5.4, which quenched all the fluorescence indicating that SEP allows the specific visualization of surface receptors^3^,^58^. Images were acquired over a period of 15 min (every 5 min). Fluorescence intensity was measured using ImageJ software only at selected regions of interest (ROI; i.e. spines) and corrected for background noise, Δ*F*/*F*^0^ was then calculated and plotted.

### Data presentation and statistical analysis

Quantification of WB analysis was performed by means of computer-assisted imaging (ImageJ) after normalization on Tubulin levels. For the original WB, please see Supplementary Fig. 6. For surface/total ratio assays, co-localization and morphological analysis, cells were chosen randomly for quantification from four to eight different coverslips (two to three independent experiments), images were acquired using the same settings/exposure times, and at least ten cells for each condition were analysed. Co-localization analysis was performed using AIM 4.2 software (Zeiss). Surface ratios were obtained by dividing the background subtracted fluorescence intensities measured with ImageJ. Morphological analysis was performed with ImageJ software to measure spine head width.

All the group values are expressed as mean±s.e.m. Comparisons between groups were performed using the following tests as appropriate: two-tailed unpaired Student's *t* test, Mann–Whitney test, Wilcoxon test or one-way ANOVA followed by Tukey *post-hoc* test. Significance levels were defined as *P<0.05, **P<0.01, ***P<0.001. Statistical analyses were performed using the GraphPad Prism statistical package (GraphPad software). Sample sizes are similar to those generally employed in the field for any specific type of experiment presented in the manuscript. When appropriate, experiments were performed in blind conditions. No outlining sample exclusion criteria was used.

## Additional information

**How to cite this article:** Stanic, J. *et al.* Rabphilin 3A retains NMDA receptors at synaptic sites through interaction with GluN2A/PSD-95 complex. *Nat. Commun.* 6:10181 doi: 10.1038/ncomms10181 (2015).

## Supplementary Material

Supplementary Information Supplementary Figures 1-6
