# Supplementary material for: Rabphilin 3A retains NMDA receptors at synaptic sites through interaction with GluN2A/PSD-95 complex
Source: Nat Commun. 2015 Dec 18;6:10181. doi: 10.1038/ncomms10181 (PMC4703873; doi:10.1038/ncomms10181)
Supplement: Supplementary Information — Supplementary Figures 1-6 [file ncomms10181-s1.pdf]

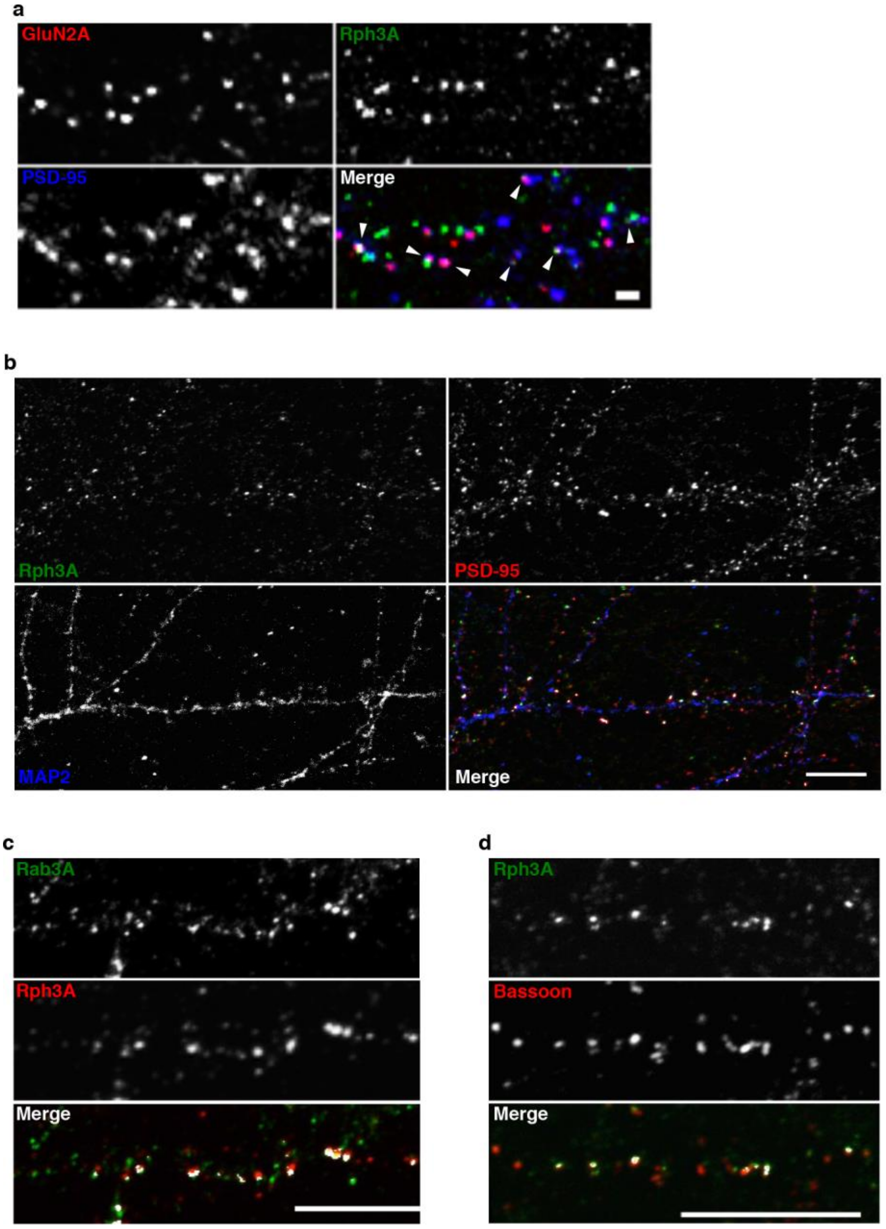

Supplementary Figure 1. Rph3A colocalizes with presynaptic partner Rab3A. (a) Fluorescence immunocytochemistry of GluN2A (red), Rph3A (green) and PSD-95 (blue) in *DIV15* primary hippocampal neurons. Arrowheads point to co-localized puncta. Scale bar: 1 µm. (b) Fluorescence immunocytochemistry of PSD-95 (red), Rph3A (green) and MAP2 (blue) in *DIV15* primary hippocampal neurons. Scale bar: 10 µm. (c) Fluorescence immunocytochemistry of Rab3A (green) and Rph3A (red) in *DIV15* primary hippocampal neurons. Scale bar: 10 µm. (d) Fluorescence immunocytochemistry of Rph3A (green) and Bassoon (red) in *DIV15* primary hippocampal neurons. Scale bar: 10 µm.

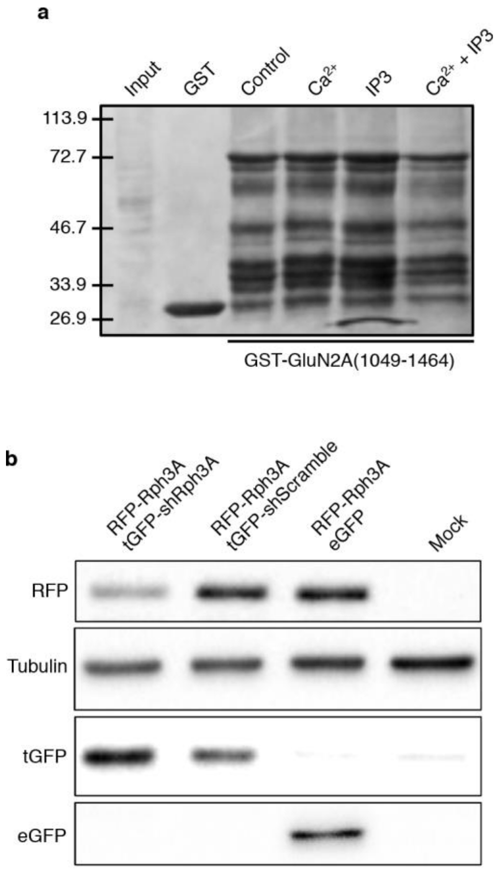

Supplementary Figure 2. (a) Ponceau staining of the input and GST-GluN2A(1049-1464) used in Figure 2c. (b) Western blotting analysis for RFP, Tubulin, turbo GFP (tGFP) and eGFP from cells lysates of COS7 cells transfected with RFP-Rph3A in presence of tGFP-shRph3A, tGFP-shScramble or eGFP.

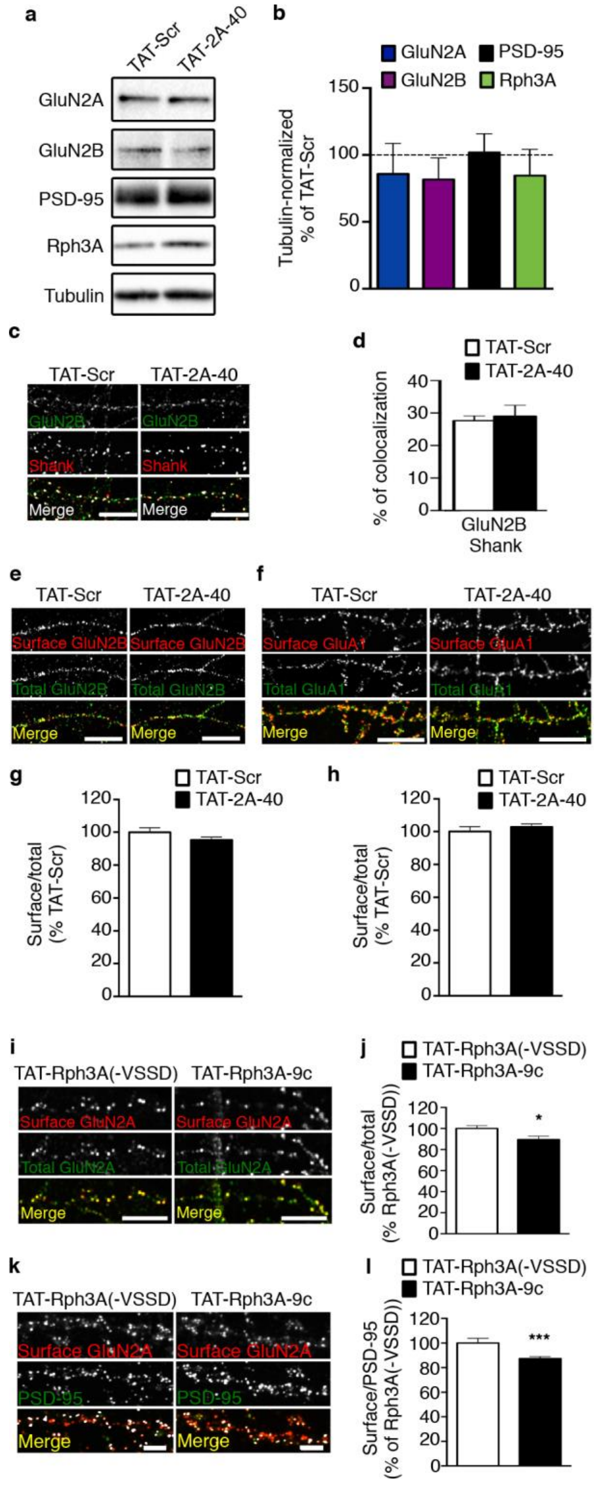

Supplementary Figure 3. **Effect of GluN2A/Rph3A/PSD-95 complexes on GluN2A synaptic availability in hippocampal neurons.** (a) WB analysis for GluN2A, GluN2B, PSD-95, Rhp3A and Tubulin performed from homogenates of *DIV15* neurons treated with TAT-Scr or TAT-2A-40 10 μM 30min. (b) The bar graph shows GluN2A, GluN2B, PSD-95 and Rhp3A protein levels in TAT-2A-40 treated samples expressed as % of TAT-scramble, n=5. (c) Fluorescence immunocytochemistry of GluN2B (green) and Shank (red) in DIV15

neurons treated with TAT-Scr or TAT-2A-40, both 10µM 30min. Scale bars: 10 µm. (d) Bar graph representing the percentage of colocalization of GluN2B with Shank, n=7-14. (e,f) Fluorescence immunocytochemistry of surface GluN2B (c, red) or GluA1 (e, red) and total GluN2B (c, green) or GluA1 (e, green) in DIV15 hippocampal neurons treated for 30 min with TAT-Scr or TAT-2A-40, both 10 µM. Scale bars: 10 µm. (g, h) Bar graph representing the percentage of integrated density ratio GluN2B (g) or GluA1 (h) surface/total compared to the mean of TAT-Scr, n=53-58 (g) and n=39-51 (h). (i) Fluorescence immunocytochemistry of surface GluN2A (red) and total GluN2A (green) in *DIV15* hippocampal neurons treated for 30 min with TAT-Rph3A(-VSSD) or TAT-Rph3A-9c, both 10 µM. Scale bars: 10 µm. (j) Bar graph representing the percentage of integrated density ratio GluN2A surface/total compared to the mean of TAT-Rph3A(-VSSD), n=33. (k) Fluorescence immunocytochemistry of surface GluN2A (red) and PSD-95 (blue) in DIV15 hippocampal neurons treated for 30 min with TAT-Rph3A(-VSSD) or TAT-Rph3A-9c, both 10 µM. Scale bars: 10 µm. (l) Bar graph representation of the percentage of integrated density ratio surface GluN2A/PSD-95 compared to the mean of TAT-Rph3A(-VSSD), n=51-100. All data are represented as mean ± SEM. *p<0.05, **p<0.01, ***p<0.001.

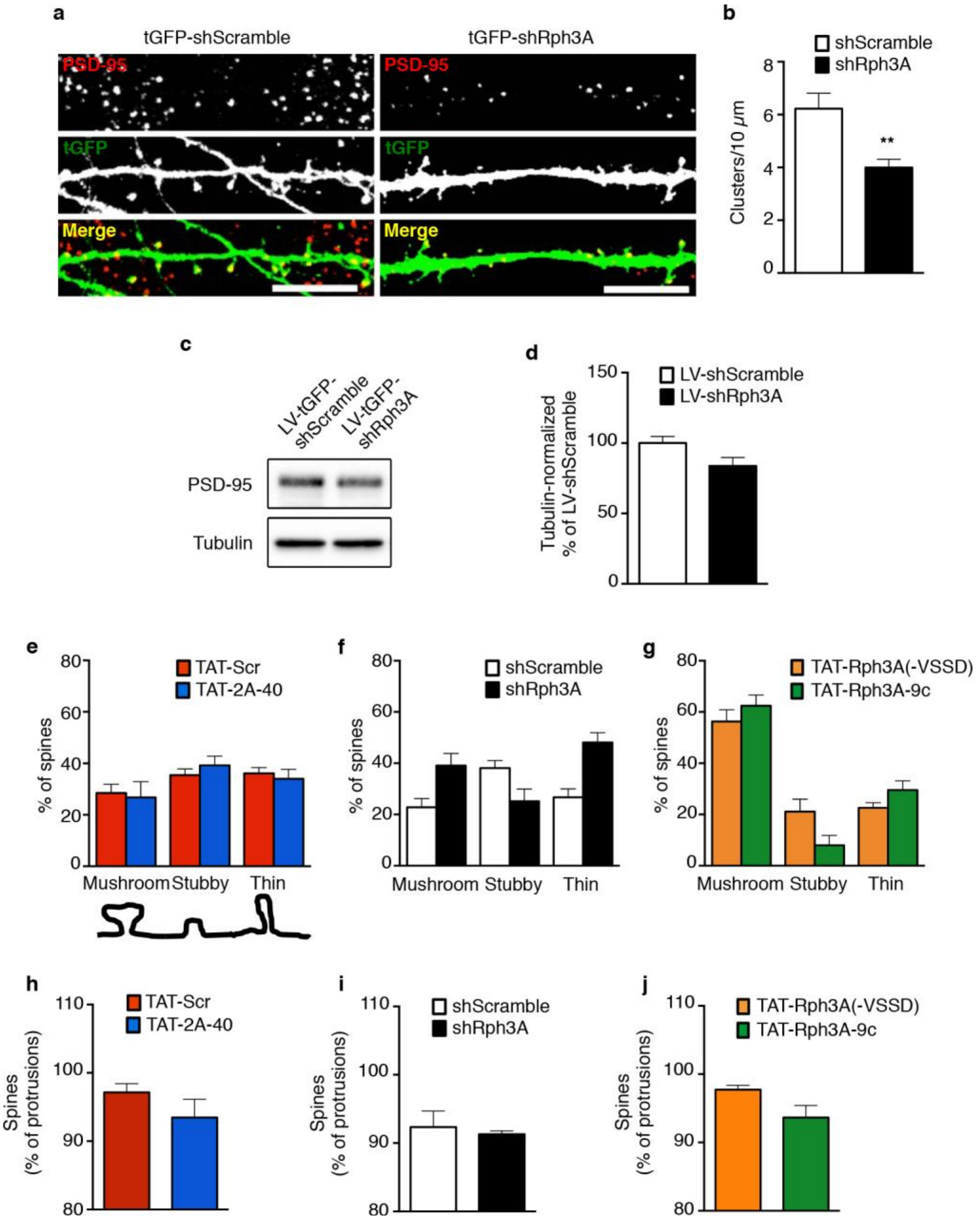

Supplementary Figure 4. Disruption of Rph3A/GluN2A interaction does not affect AMPAR EPSCs or spine morphology.

(a) Dendrite of hippocampal neuron transfected with tGFP-shScramble or tGFP-shRph3A and immunolabeled for tGFP (green) and PSD-95 (red). Scale bars: 10 μm. (b) The bar graph represents the number of PSD-95 positive clusters along 10 μm of dendrite; $^{**}p<0.01$, n=10-12. (c) WB analysis performed from lysates of *DIV15* hippocampal neurons infected with LV-tGFP-shScramble or LV-tGFP-shRph3A lentivirus (*DIV10*). (d) The graph shows PSD-95 protein levels in neurons transfected with tGFP-shRph3A compared with tGFP-shScramble (n=6). (e-g) Bar graph representation of the different spine types percentage compared to the total spines (mushroom, stubby and thin) of primary hippocampal neurons treated with either TAT-Scr or TAT-2A-40 (c), TAT-Rph3A(-VSSD)

or TAT-Rph3A-9c (e) or transfected with shScramble or shRph3A (d), n=5. All data are presented as mean ± SEM.

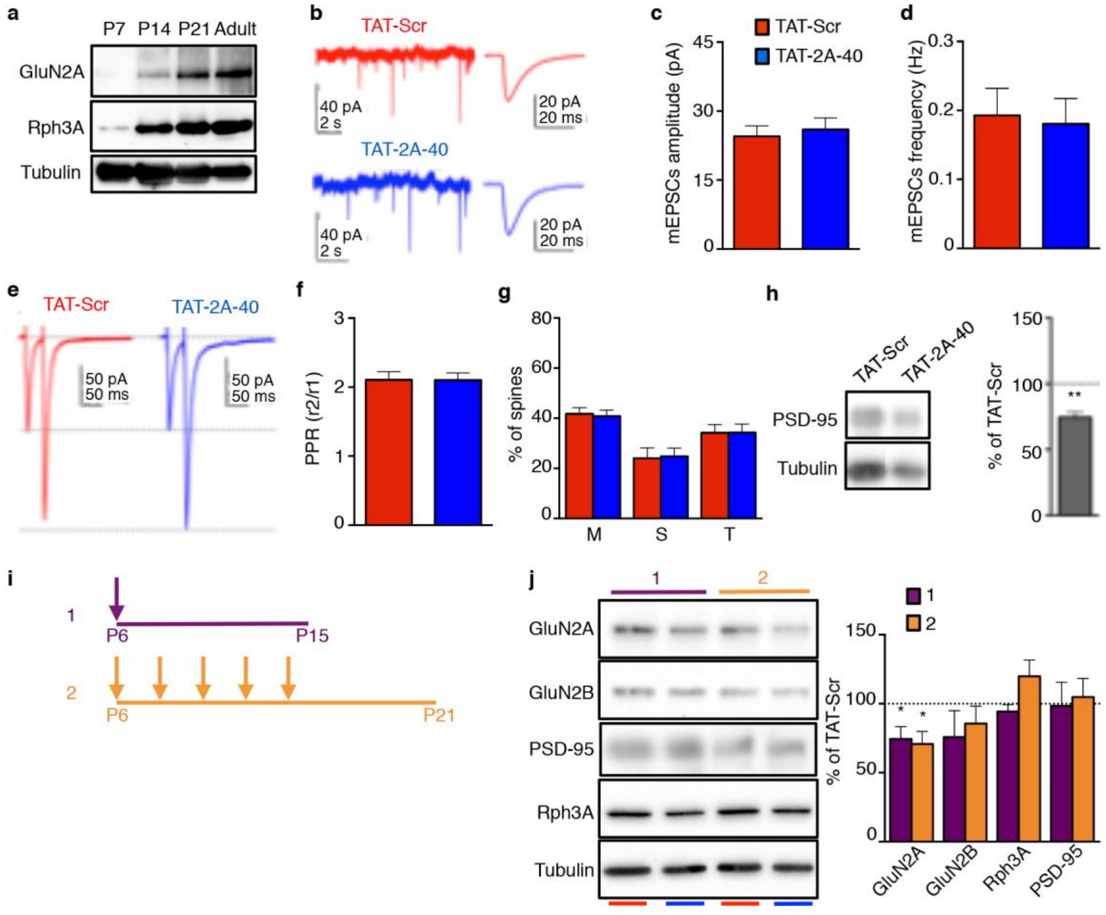

Supplementary Figure 5. Modulation of GluN2A-containing NMDAR expression at synapses in the developing rat hippocampus. (a) Western blot analysis of GluN2A, Rph3A, PSD-95 and Tubulin of brain homogenates from P7, P14, P21 and Adult rats. (b) Sample traces of mEPSCs recorded from CA1 pyramidal cells in acute slices prepared from TAT-Scr treated (red) or TAT-2A-40 treated animals (blue). On the right average mEPSCs traces, with a scale of 20 pA over 20 ms, obtained from the recording shown on the left, with a scale of 40 pA over 2 s. (c, d) Bar graphs summarizing that the amplitude (c) and the frequency (d) of the mEPSCs did not change between TAT-Scr treated (red) or TAT-2A-40 treated animals (blue), n = 7-8. (e) Sample traces of paired pulse responses (40 ms interval, average of 8-12 traces) recorded from CA1 pyramidal cells while stimulating Schaffer collaterals with a scale of 50 pA over 50 ms. (f) Bar graphs summarize that PPR (response 2 / response 1) did not change between TAT-Scr treated (red) or TAT-2A-40 treated animals (blue), n=9-11. (g) Bar graph representation of the different spine types percentage compared to the total spines (mushroom, stubby and thin) of P15 pups treated chronically from P6 to P14 with either TAT-Scr or TAT-2A-40. (h) Western blot analysis of PSD-95 and tubulin performed from the homogenate obtained from

hippocampus of chronically treated P15 rat pups. The bar graph represents the percentage of Tubulin normalized integrated density of PSD-95 WB bands from homogenate samples compared to their respective TIF purification TAT-Scr control, n=2-5. (i, j) Illustration of experimental design, arrows represent injection time point of TAT-Scr or TAT-2A-40 3 nmol/g s.c. 1 (purple), single injection at P6 and animals were sacrificed at P15; 2 (orange), 5 chronic injections at P6, P8, P10, P12 and P14 and animals were sacrificed at P21 (i) and western blot analysis of GluN2A, GluN2B, PSD-95, Rph3A and Tubulin performed from the Triton insoluble postsynaptic fraction (TIF) obtained from hippocampus of treated rat pups (j). Red bars represent TAT-Scr treated animals, blue bars represent TAT-2A-40 treated animals. The bar graph represents the percentage of Tubulin normalized integrated density of GluN2A, GluN2B, Rph3A and PSD-95 WB bands from TIF samples compared to their respective TIF purification TAT-Scr control, n=3. All data are presented as mean ± SEM. *$p<0.05$, **$p<0.01$, ***$p<0.001$.

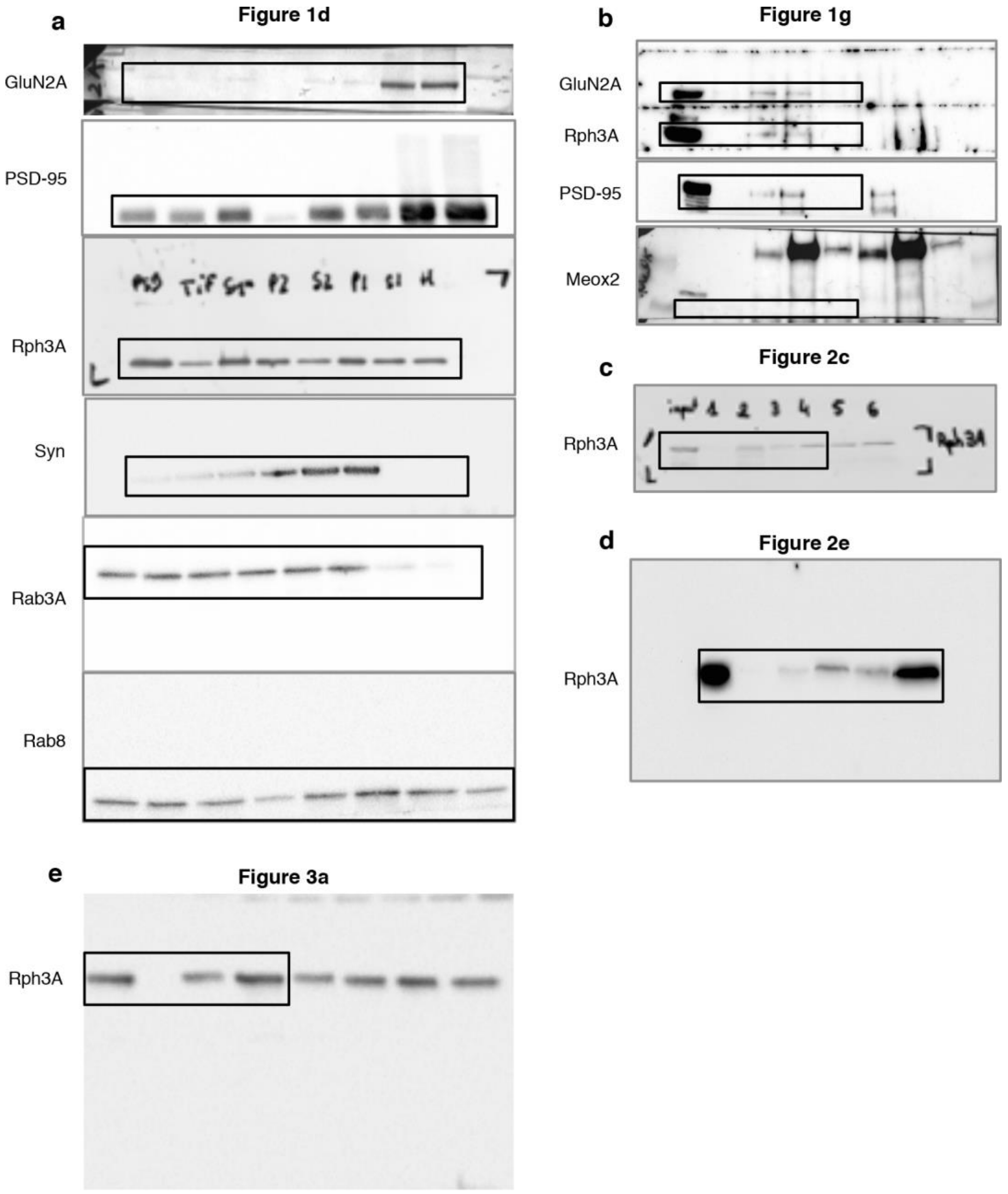

Supplementary Figure 6: Images of full-length blots presented in main Figures. (a) Uncropped version of western blots presented in Figure 1d. (b) Uncropped version of western blots presented in Figure 1g. (c) Uncropped version of western blots presented in Figure 2c. (d) Uncropped version of western blots presented in Figure 2e. (e) Uncropped version of western blots presented in Figure 3a.

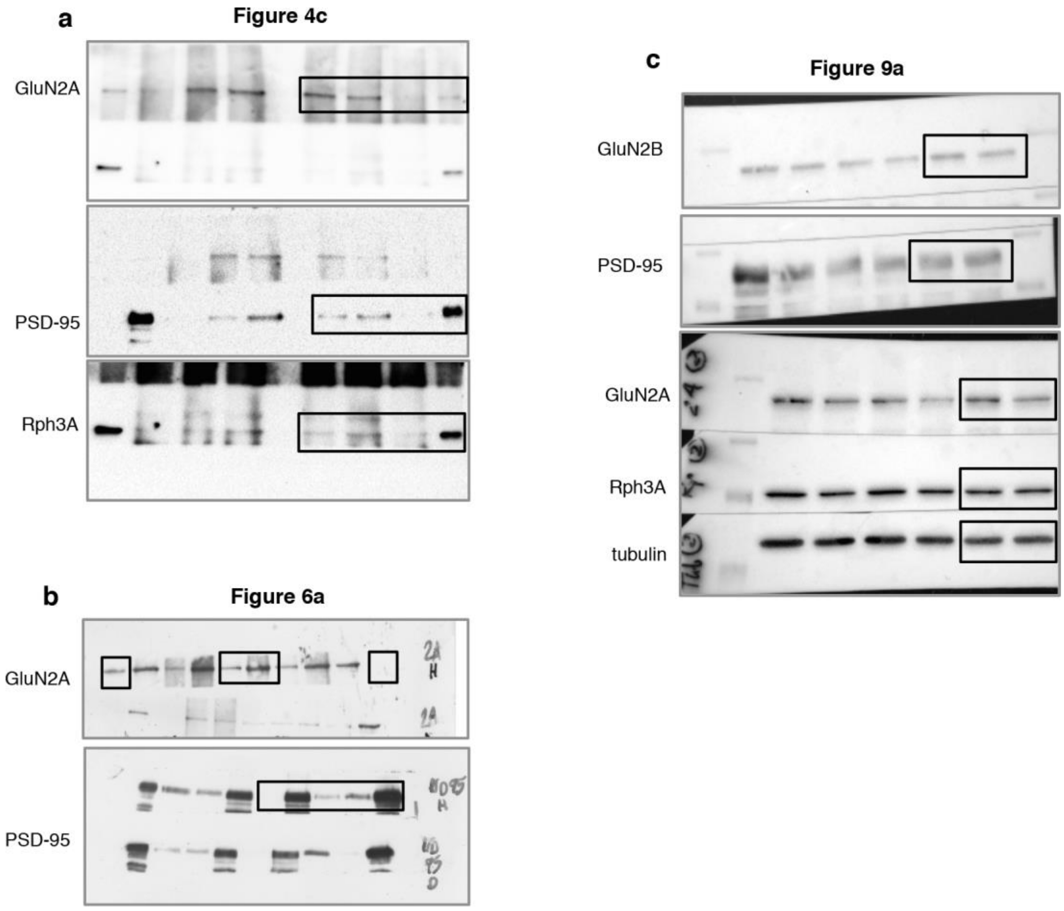

Supplementary Figure 6 (continued): Images of full-length blots presented in main Figures. (a) Uncropped version of western blots presented in Figure 4c. (b) Uncropped version of western blots presented in Figure 6a. (c) Uncropped version of western blots presented in Figure 9a.
